# Synthesis, Structures and Corrosion Inhibition Properties of 4-Nitrophenylacetato-Rare-Earth(III) 1D Coordination Polymers

**DOI:** 10.3390/molecules30193940

**Published:** 2025-10-01

**Authors:** Jacob M. Neill, Naveena Y. Salpadoru Thuppahige, Zhifang Guo, Glen B. Deacon, Peter C. Junk

**Affiliations:** 1 College of Science & Engineering, James Cook University, Townsville, QLD 4811, Australia; jacob.neill1@my.jcu.edu.au (J.M.N.);; 2 School of Chemistry, Monash University, Clayton, VIC 3800, Australia

**Keywords:** rare earth, carboxylate complexes, 4-nitrophenylacetate, X-ray structures, corrosion inhibitors

## Abstract

The rare earth (RE) aqua 4-nitrophenylacetate (4npa) complexes {[RE(4npa)_3_(H_2_O)_2_]·2H_2_O}_n_ (RE = La (**1La**), Nd (**2Nd**)), [Ce(4npa)_3_(H_2_O)_2_]_n_ (**3Ce**), and {[RE_2_(4npa)_6_(H_2_O)]·2H_2_O}_n_ (RE = Gd (**4Gd**), Dy (**5Dy**), Y (**6Y**), Er (**7Er**), Yb (**8Yb**)) were synthesised by salt metathesis reactions of RE^III^ chlorides or nitrates with sodium 4-nitrophenylacetate Na(4npa) in aqueous ethanol. The structures of all the complexes were determined by single-crystal X-ray diffraction (SCXRD) except for RE = **4Gd**, which was determined to be isomorphous with the **5Dy** and **7Er** complexes by X-ray powder diffraction (XRPD). All the complexes crystallise as one-dimensional polymers linked by bridging carboxylates. Complexes (**1La**–**3Ce**) have mononuclear repeating units with two coordinated waters and ten coordinate RE ions, **1La** and **2Nd** also have two waters of crystallization, but **3Ce** has none. By contrast, complexes (**4Gd**–**8Yb**) have binuclear repeating units with a single coordinated water. Isomorphous **5Dy** and **7Er** have one nine coordinate and one eight coordinate metal ion, whilst isomorphous **6Y** and **8Yb** have two eight coordinate RE ions. In some cases, bulk powders have structures different from the corresponding single crystals. For example, bulk **1La** is isomorphous with **3Ce** owing to the loss of water of crystallization, and **8Yb** exhibits coordination isomerism between single crystals and microcrystalline powder. Weight loss corrosion tests revealed that {[Dy_2_(4npa)_6_(H_2_O)]·2H_2_O}_n_ (**5Dy**) has the greatest inhibition efficiency (89%) of the complexes (**1La**–**8Yb**). The activities are comparable to those of the corresponding 4-hydroxyphenylacetates (4hpa) and far superior to those of 2-hydroxyphenylacetates (2hpa) and the unsubstituted phenylacetates. Whilst the coordination numbers generally decline with the lanthanoid contraction, there are deviations around **5Dy**, **6Y**, **7Er**, and **8Yb**, and the corrosion inhibition is optimised with a midrange size.

## 1. Introduction

One of the attractive features of rare earth carboxylate coordination chemistry is the variety of their structures [1]. This is enabled by the numerous possible carboxylate coordination modes [2], the capacity of carboxylate complexes to take part in supramolecular interactions [3], and the high coordination numbers and non-directional bonding exhibited by rare earth ions [4,5]. Thus, most groups of rare earth carboxylates reveal novel and interesting structural features. Amongst the possible and actual applications of rare earth carboxylates [6] are their use as corrosion inhibitors [7,8].

The corrosion of metals is an electrochemical process resulting in oxidative degradation of the metal surface, and it can be countered by the use of corrosion inhibitors [7,8,9]. Historically, methods of metal corrosion inhibition for steel and aluminium have used chromates, which are known to be toxic, with the potential to cause DNA damage [10,11]. More recently, benzoates, nitrites, oxalates and phosphates have been used for the passivation of steel [12,13,14]. Developments in the use of rare earth arenecarboxylates have provided a much less toxic alternative to traditional chromate use, allowing inhibition of both the anodic and cathodic reactions [8,15]. Alternative approaches to corrosion inhibition include coatings such as a red iron oxide epoxy coating [16], a molecular assembly between a cyclodextrin polymer and trans-cinnamaldehyde [17], a multilayer TiO_2_ coating [18], lignin based ionic liquids [19], and multifunctional inhibitor coatings [20], whilst the use of polymeric corrosion inhibitors has been reviewed [21].

**Figure 1:**
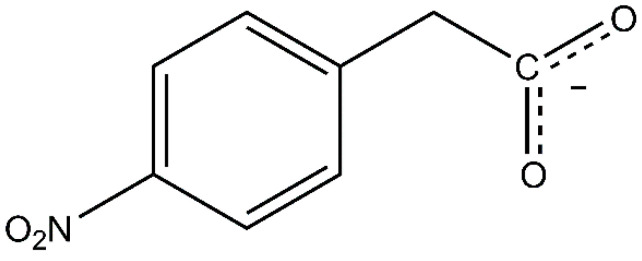
4-nitrophenylacetate (4npa).

The use of rare earth arene carboxylates began with the finding that cerium salicylate is as an effective corrosion inhibitor [22], and attention then turned to lanthanoid cinnamates, where it was found that lanthanum 4-hydroxycinnamate is a superior inhibitor compared to lanthanum cinnamate [23]. The enhancement produced by the 4-hydroxy substituent can be attributed to the attachment of the inhibitor to the surface by condensation of the substituent with a surface Fe-OH to give an Fe-O-Ar linkage [24] and to direct ArOH-Fe coordination. To further illustrate the effects of substituents on corrosion inhibition, adding a 4-hydroxy substituent to lanthanoid phenylacetates, which have indifferent anticorrosion properties [25], greatly enhanced the anti-corrosion behaviour [24]. In the initial study of lanthanoid cinnamates, it was found that lanthanum 4-nitrocinnamate was essentially as effective as 4-hydroxycinnamate [23]. This was not followed up despite the interesting contrast in electronic substituent effects, with the hydroxy electron donating by resonance, whereas the nitro is electron withdrawing both inductively and by resonance. In the present study, we examined the synthesis and structures of a series of lanthanoid 4-nitrophenylacetate (4npa) complexes to see if the nitro substituent can cause a significant inhibitory enhancement in this challenging test system. From a structural viewpoint, 4npa (Figure 1) is quite a different ligand from 4hpa, as the latter is primed for hydrogen bonding as a donor, as is evident in the rare earth complexes [24], whereas the former could be an H-bond acceptor and the nitro group is known to coordinate to rare earths [26,27]. What both substituents have in common is the capacity to coordinate, providing a handle to attach to a steel surface. Thus, 4npa presents interesting coordination potential in rare earth coordination, and we have prepared RE metal (Y, La, Ce, Nd, Gd, Dy, Er, Yb) complexes with 4npa and determined their structures and corrosion inhibition properties.

4npa metal complexes are known for smaller metal centres in distorted octahedral arrangements, as in [M(4npa)_2_(μ_2_-H_2_O)(H_2_O)_2_]·2H_2_O [28] (M = Mn, Co, and Zn). Additionally, 4npa compounds have been synthesised and characterised for alkaline earth metals: Mg, Ca, and Sr, in [Mg(H_2_O)_6_](4npa)_2_·4H_2_O, [Ca(H_2_O)_2_(4npa)_2_], and [Sr(H_2_O)_3_(4npa)_2_]·4.5H_2_O, in which 4npa is a counter ion [29]. Only one rare earth npa complex has been structurally characterized, viz [Tb(npa)_3_(H_2_O)(dmso)_2_] [30], which is a 1D polymer based on a single Tb atom repeating unit.

## 2. Results and Discussion

### 2.1. Synthesis and Characterisation

**Scheme 1:**
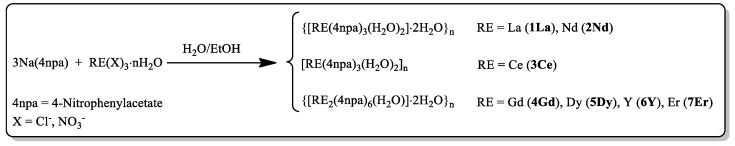
Salt metathesis reaction for the preparation of RE^III^ carboxylates.

RE^III^ 4npa complexes were synthesised by a simple metathesis reaction in which the Na salt of the ligand was added to hydrated RE^III^ metal nitrates or chlorides in a 3:1 molar ratio (Scheme 1).

Characterisation of each compound was performed by X-ray powder diffraction (XRPD), single crystal X-ray diffraction (SCXRD), thermogravimetric analysis (TGA), melting point, and attenuated total reflectance Fourier transform infrared (ATR-FTIR) spectroscopy, as well as complexometric titration with ethylenediaminetetraacetic acid (EDTA) for metal analysis [31,32,33], and microanalysis. A large amount of precipitate was observed for each of the complexes owing to the general insolubility of the RE^III^ 4npa complexes in both polar and non-polar solvents (for 4npaH solubility [34]). The solubility of each complex in water was found to be in the range 1300–3200 ppm (1.3 g/L–3.2 g/L). The compositions were obtained from structural determinations by SCXRD and were generally in agreement with the elemental analyses. The first step in the TGA weight loss corresponded to the loss of all water (coordinated and water of crystallization) except in the case of **2Nd**, the powder of which had already undergonesubstantial weight loss (−3H_2_O) before being tested. The second step in the weight loss, which does not provide structural information, is discussed in the Supplementary Information (Table S10 and related text).

The ν(OH) absorptions of the aqua complexes at 3600–3150 cm^−1^ are very broad and poorly resolved (Figures S1 and S3–S10), especially as seven of the complexes also have water of crystallization, hence, any variations in the apparent peak positions are without significance. The strong peak present at ~1700 cm^−1^ associated with carboxylic acid C=O stretching is absent in the IR spectra of the lanthanoid complexes. These shifts are indicative of deprotonation of the carboxylic acid and coordination to the RE^III^ metal. Strong NO_2_ stretching absorptions are observed at 1510–1504 and 1343–1338 cm^−1^ for each compound without much deviation from the free acid values (1508, 1339 cm^−1^) and are consistent with values expected for *p*-nitroarene species [35], suggesting no involvement in coordination. Asymmetric and symmetric CO_2_ stretching is observed at 1576–1541 and 1401–1386 cm^−1^, respectively, and the relatively small separation (Table 1) is as expected for the chelating and bridging coordination modes observed in the structures [36].

### 2.2. X-Ray Crystal Structures

**Figure 2:**
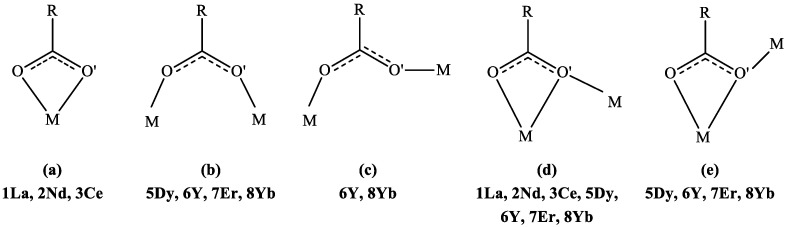
Each of the coordination modes of 4-nitrophenylacetate observed in the structures of complexes **1La**–**8Yb**: (**a**) *syn*-*syn* chelate—1κ(O,O′); (**b**) *syn*-*syn* bridging bidentate—μ-1κ(O)-2κ(O′); (**c**) *syn*-*anti* bridging bidentate—μ-1κ(O)-2κ(O′); (**d**) *syn*-*syn* chelating bridging tridentate—μ-1κ(O)2κ(O,O′); and (**e**) *syn*-*anti* chelating bridging tridentate—μ-1κ(O)2κ(O,O′).

The coordination modes of 4-nitrophenylacetate complexes **1La**–**8Yb** are shown in Figure 2. Crystal data and structural refinements, selected bond lengths, bond angles and hydrogen bond distances can be found in the Supplementary Information for complexes **1La**–**8Yb** (Tables S1–S9).

#### 2.2.1. Complexes {[RE(4npa)_3_(H_2_O)_2_]·2H_2_O}_n_ (RE = La (**1La**), Nd (**2Nd**))

**Figure 3:**
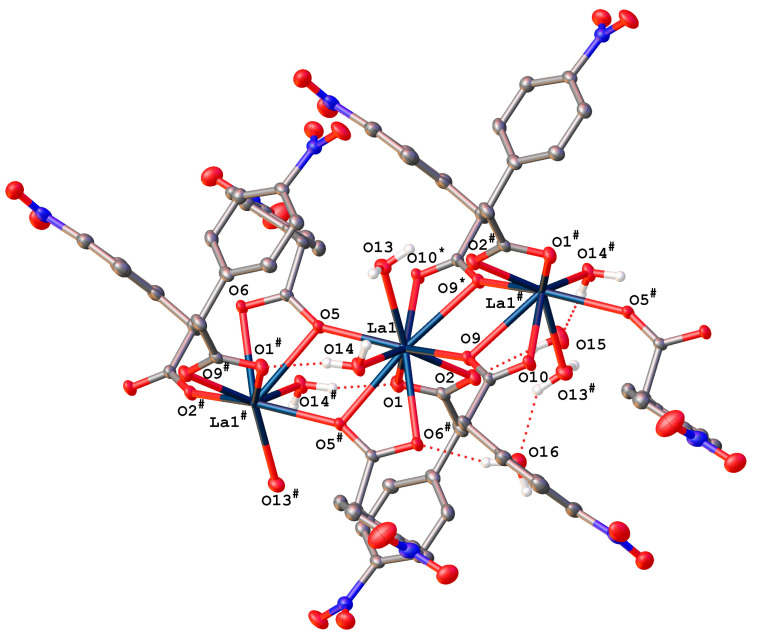
A section of the polymer of {[La(4npa)_3_(H_2_O)_2_]·2H_2_O}_n_ (**1La**) (representative of La (**1La**), Nd (**2Nd**)) with 50% thermal ellipsoids. Dotted lines indicate the inter-molecular hydrogen bonds. Hydrogen atoms have been omitted for clarity except in the case of H-bond contribution and coordinated water. Selected bond lengths (Å) for (**1La**): (data for (**2Nd**) in Supplementary Information): La1-O1 2.5871(8), La1-O2 2.6100(6), La1-O5 2.4924(8), La1-O9 2.4931(8), La1-O9* 2.6900(8), La1-O10* 2.5591(8), La1-O5^#^ 2.6938(10), La1-O6^#^ 2.6135(12), La1-O13 2.6003(9), and La1-O14 2.5895(6). Symmetry code: #1-*x*,1-*y*,1-*z*; *2-*x*,1-*y*,1-*z*.

The first structural type {[RE(4npa)_3_(H_2_O)_2_]·2H_2_O}_n_ (RE = La (**1La**), Nd (**2Nd**)) complexes crystallise in the triclinic P$\bar{1}$ space group as one-dimensional polymers with 10-coordinate bicapped square antiprismatic Ln^III^ metal ions (Figure 3). The complexes are isomorphous and are similar in structure to **3Ce**, varying by **1La** and **2Nd** having waters of crystallisation. In the asymmetric unit, the La1 atom is bound by three chelating 4npa ligands, two also bridging μ-1κ(O)2κ(O,O′), (O9*-La1-O10* 0.131 Å difference, O5^#^-La1-O6^#^ 0.080 Å difference), and a single chelating 1κ(O,O′) 4npa ligand (O1,O2 0.023Å difference). In addition, La1 is bridged in the polymer by O5 and O9 from the O5,O6 and O9,O10 ligands which chelate adjacent metal centres. The complexes also have two ligated *cisoid* water molecules (O13-La1-O14 68.86(3)) and two waters of crystallisation (O15,O16). One ligated water molecule exhibits hydrogen bonding to the oxygen of an adjacent 4npa ligand (H14A···O1^#^), with a bond length of 1.8625(5) Å, and both ligated waters exhibit hydrogen bonding to adjacent lattice waters H14B^#^···O15, H13*A···O16, with bond lengths of 1.8877(6) and 2.0212(9) Å, respectively. Each lattice water exhibits hydrogen bonding to oxygen from adjacent 4npa ligands, with a bond length of 1.878(14) Å and 1.9263(13) Å for H15A···O2 and H16A···O6^#^, respectively (Table S3). The distances between adjacent La1^#^-La1 and La1-La1* metal ions are 4.4081(9), and 4.3197(9) Å, respectively. The bond angle (La1-O5(or O5^#^)-La1^#^ at the bridging oxygen atoms O5,O5^#^ is 116.36(2). The bond angle (La1-O9(or O9*)-La1* at the bridging oxygen atoms O9,O9* is 112.85(19). The average La1-O bond length of chelating oxygens is 2.63 Å, with an average bond length of 2.49 Å for bridging oxygen atoms (Figure 3).

#### 2.2.2. Complex [Ce(4npa)_3_(H_2_O)_2_]_n_ (**3Ce**)

**Figure 4:**
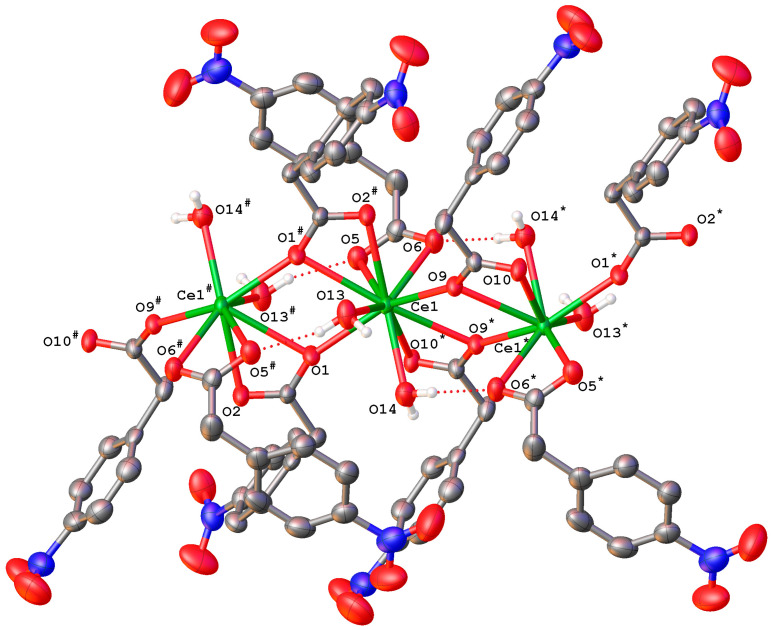
A section of the polymer [Ce(4npa)_3_(H_2_O)_2_]_n_ (**3Ce**) with 50% thermal ellipsoids. Dotted lines indicate the inter-molecular hydrogen bonds. Hydrogen atoms have been omitted for clarity except in the case of H-bond contributions and coordinated water. Selected bond lengths (Å) for (**3Ce**): Ce1-O1 2.43231(6), Ce1-O1^#^ 2.75646(8), Ce1-O2^#^ 2.56551(7), Ce1-O5 2.57343(6), Ce1-O6 2.56302(8), Ce1-O9 2.45223(7), Ce1-O9* 2.69219(8), Ce1-O10* 2.57034(9), Ce1-O13 2.62747(7), and Ce1-O14 2.63829(8). Symmetry code: #1-*x*,1-*y*,1-*z*; *2-*x*,1-*y*,1-*z*.

The second structural type [Ce(4npa)_3_(H_2_O)_2_]_n_ (**3Ce**) crystallises in the triclinic P$\bar{1}$ space group as a one-dimensional polymer with a 10-coordinate bicapped square antiprismatic Ce metal ion (Figure 4). In the asymmetric unit, the Ce metal is bound by three chelating 4npa ligands. Two are tridentate *syn*-*syn* μ-1κ(O,O′):2κ(O′) 4npa ligands, which chelate O1^#^,O2^#^ (O1^#^-Ce1-O2^#^ 0.191 Å difference) and O10*,O9* (O10*-Ce1-O9* 0.122 Å difference) and bridge adjacent metal centres in the polymer. The asymmetric unit also has a single chelating 1κ(O,O′) 4npa ligand (O5,O6), two bridging oxygens (O1,O9) and two ligated *cisoid* water molecules (O13,O14), completing the ten coordination of Ce1. The two ligated water molecules exhibit hydrogen bonding to the oxygens of adjacent 4npa ligands (H14A···O6*) and (H13A···O5^#^) of 1.89229(6) Å and 1.9393(6) Å, respectively (Table S5). The distances between adjacent Ce1^#^-Ce1 and Ce1-Ce1* metal ions are 4.38277(15), and 4.32248(15) Å, respectively. The bond angle of Ce1-O1(or O1^#^)-Ce1^#^ is 115.12(2), and between Ce1-O9(or O9*)-Ce1* it is 114.248(3). The average Ce1-O_carboxylate_ bond length of chelating oxygens is 2.62 Å, with an average (shorter) bond length of 2.44 Å for bridging oxygen atoms in the tridentate bridging (μ-1κ(O)2κ(O,O′)) ligands.

#### 2.2.3. Complexes {[RE_2_(4npa)_6_(H_2_O)]·2H_2_O}_n_ (RE = Dy (**5Dy**), Er (**7Er**))

**Figure 5:**
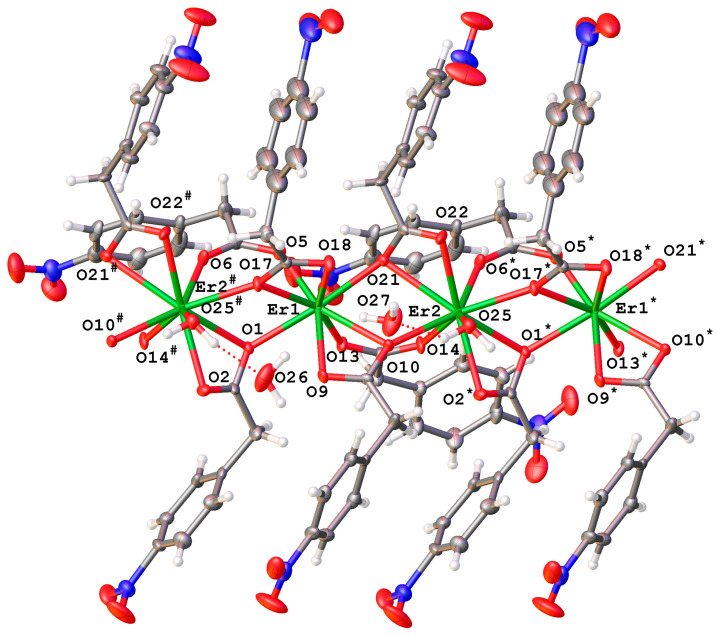
A section of the polymer of {[Er_2_(4npa)_6_(H_2_O)]·2H_2_O}_n_ (**7Er**) (representative of Dy (**5Dy**), Er (**7Er**)) with 20% thermal ellipsoids. Dotted lines indicate the inter-molecular hydrogen bonds. Hydrogen atoms have been omitted for clarity except in the case of H-bond contributions and coordinated water. Selected bond lengths (Å) for (**7Er**): (data for (**5Dy**) in Supplementary Information): Er1-O1 2.31051(13), Er1-O5 2.25528(12), Er1-O9 2.45140(15), Er1-O10 2.41049(14), Er1-O13 2.23180(17), Er1-O17 2.42417(15), Er1-O18 2.4767(2), Er1-O21 2.30836(14), Er2-O10 2.35117(15), Er2-O14 2.2975(2), Er2-O21 2.61648(16), Er2-O22 2.38528(14), Er2-O25 2.3788(2), Er2-O1* 2.55105(5), Er2-O2* 2.39917(17), Er2-O6* 2.29074(13), and Er2-O17* 2.36089(14). Symmetry code: #-1+*x*,+*y*,+*z*, *1+*x*,+*y*,+*z*.

Complexes of the third structural type {[RE_2_(4npa)_6_(H_2_O)]·2H_2_O}_n_ (RE = Dy (**5Dy**), Er (**7Er**)) crystallise in the monoclinic *P*2_1_ space group as binuclear one-dimensional polymers with a difference in coordination numbers between Er1 and Er2 atoms, which have distorted square antiprismatic 8-coordination and slightly distorted tricapped trigonal prismatic 9-coordination, respectively (Figure 5). In the asymmetric unit, the Er metals are coordinated by six bridging 4npa ligands. Er1 is bound to all six 4npa ligands, chelated by two tridentate *syn*-*syn* μ-1κ(O,O′):2κ(O′) 4npa ligands bound through O9,O10 and O17,O18 (O9-Er1-O10 0.041 Å difference and O17-Er1-O18 0.053 Å difference), O10 bridges to the adjacent Er2 metal centre and O17 bridges to Er2^#^ in the polymer chain. Additionally, Er1 is also bound by the oxygens (O1 and O21) of two additional *syn*-*syn* μ-1κ(O,O′):2κ(O′) 4npa ligands, which chelate an adjacent Er2^#^ or Er2 (O1-Er2^#^-O2 0.152 Å difference, O21-Er2-O22 0.231 Å difference) and oxygens (O5,O13) from two *syn*-*syn* μ-1κ(O)-2κ(O′) 4npa ligands. Er2 is chelated (O21,O22 and O1*,O2*) by two tridentate *syn*-*syn* μ-1κ(O,O′):2κ(O′) ligands, which bridge through O1* and O21 to the adjacent Er1* and Er1 atoms. Additionally, Er2 is bound by O14 and O6* from two *syn*-*syn* μ-1κ(O)-2κ(O′) ligands, O10 and O17* bridging from two *syn*-*syn* μ-1κ(O,O′)-2κ(O′) ligands, and has a single ligated water molecule (O25). The complex also has two waters of crystallisation (O26,O27). The complexes exhibit hydrogen bonding to lattice water through H25A^#^···O26 and H25B···O27, with bond lengths of 1.81399(10) and 2.03973(13) Å, respectively (Table S7). The distances between adjacent metal ions are 3.9767(3) (Er1-Er2) and 3.9763(3) Å (Er1-Er2^#^ and Er2-Er1*). The bond angles at the bridging oxygen atoms O10,O21 between Er2 and Er1 are 113.257(5) and 107.536(6), respectively. The bond angles at the bridging oxygen atoms O1,O17 between Er1 and Er2^#^ are 109.653(5) and 112.394(6), respectively. The average M-O bond length for chelated carboxylates is 2.44 Å for Er1 and 2.49 Å for Er2, with an average (shorter) bond length of 2.31 Å for Er1 bridging oxygen atoms in the tridentate bridging (μ-1κ(O)2κ(O,O′)) ligands and 2.36 Å for Er2.

#### 2.2.4. Complexes {[RE_2_(4npa)_6_(H_2_O)]·2H_2_O}_n_ (RE = Y (**6Y**), Yb (**8Yb**))

**Figure 6:**
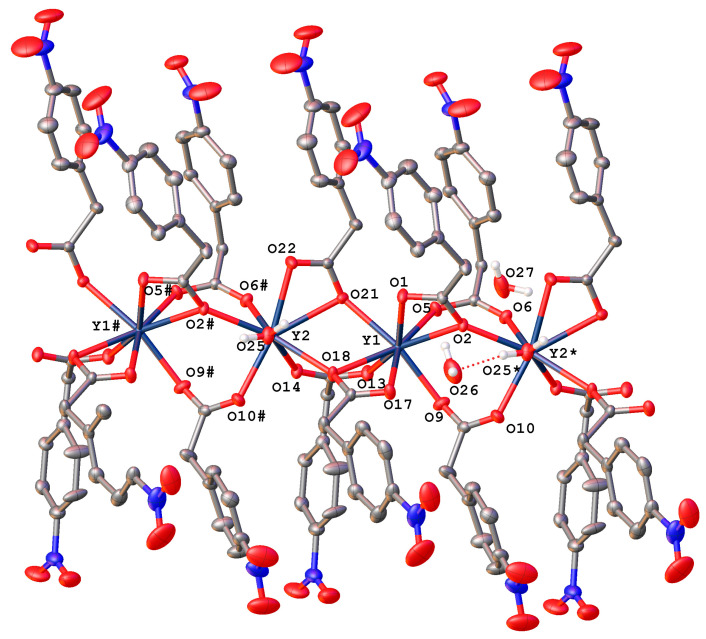
A section of the polymer of {[Y_2_(4npa)_6_(H_2_O)]·2H_2_O}_n_ (**6Y**) (also representative of Yb (**8Yb**)) with 20% thermal ellipsoids. Dotted lines indicate the inter-molecular hydrogen bonds. Hydrogen atoms have been omitted for clarity except in the case of H-bond contributions and coordinated water. Selected bond lengths (Å) for (**6Y**): (data for (**8Yb**) in Supplementary Information): Y1-O1 2.4143(12), Y1-O2 2.48063(12), Y1-O5 2.24703(11), Y1-O9 2.25021(12), Y1-O13 2.25463(11), Y1-O17 2.45972 (15), Y1-O18 2.41301(11), Y1-O21 2.36324(11), Y2-O14 2.26516(12), Y2-O18 2.4045(11), Y2-O21 2.54747(13), Y2-O22 2.42699(16), Y2-O25 2.33185(17), Y2-O2^#^ 2.38218(12), Y2-O6^#^ 2.30811(12), and Y2-O10^#^ 2.29587(13). Symmetry code: #+*x*,+*y*,1+*z*; *+*x*,+*y*,-1+*z*.

Complexes of the fourth structural type {[RE_2_(4npa)_6_(H_2_O)]·2H_2_O}_n_ (RE = Y (**6Y**), Yb (**8Yb**)) crystallise in the monoclinic *Cc* space group as binuclear based one-dimensional polymers with 8-coordinate distorted square antiprismatic metals (Figure 6). The asymmetric complex has six bridging 4npa ligands. Three bind *syn*-*syn* μ-1κ(O,O′):2κ(O′), where two (O1,O2 and O17,O18) chelate Y1 (O1-Y1-O2 0.066 Å difference, O17-Y1-O18 0.047 Å difference) and bridge to Y2* through O2 and Y2 through O18. One (O21,O22) chelates Y2 (O21-Y2-O22 0.120 Å difference) and bridges through O21 to bind adjacent Y1. The complex also has two bridging *syn*-*syn* μ-1κ(O):2κ(O′) ligands, one (O13,O14) bridging between Y1 and Y2 and one (O5,O6) bridging between Y1 and Y2*. An additional *syn*-*anti* μ-1κ(O):2κ(O′) ligand (O9,O10) bridges Y1 and Y2*. Additionally, Y2 also has a single ligated water molecule (O25), with the complex also having two waters of crystallisation (O26,O27). One ligated water molecule exhibits hydrogen bonding to the oxygen of an adjacent lattice water (H25B*···O26), with a bond length of 1.85427(9) Å (Table S9). The distances between adjacent Y2-Y1 and Y1-Y2* metal ions are 3.9850(2) and 4.2203(3) Å, respectively. The Y1-(O18)-Y2 and Y1-(O21)-Y2 bond angles are 111.623(4) and 108.426(5) Å, respectively. The bond angle subtended at the bridging oxygen atom (O2) between Y1 and Y2* is 120.41(4). The average M-O bond length for chelated carboxylates is 2.44 Å for Y1 and 2.49 Å for Y2, with an average (shorter) bond length of 2.36 Å for Y1 bridging oxygen atoms in the tridentate bridging (μ-1κ(O)2κ(O,O′)) ligands and 2.39 Å for Y2. Y, Er, and Yb have similar ionic radii of 1.02, 1.01 and 0.99 Å for eight coordination, leading to similarities in coordination behaviour [37]. However, the sequence of coordination numbers for RE(2), namely 8 (Y), 9 (Er), 8 (Yb), is contrary to lanthanoid contraction expectations. Such discontinuities are rare but not unknown, and a more striking case has been observed in the structures of some rare earth 3,5-diphenylpyrazolates [38].

A decrease in size across the lanthanoid period (lanthanoid contraction) correlated with a decrease in the observed average bond lengths, from 2.59 Å in the La complex (**1La**) through to 2.33 Å in the Yb complex (**8Yb**) (Table 2).Furthermore, two different metal atoms were observed in the asymmetric unit of smaller rare earth metals (Dy, Y, Er, Yb), but only one type of metal atom was observed for metals with larger ionic radii (La, Ce, Nd) [37]. Despite the possibility of -NO_2_-RE coordination, none was observed with -NO_2_-RE distances in the range 4.77–8.84 Å. These are well outside the sum of the appropriate van der Waals radii [39]. In addition, the -NO_2_ groups are not involved in H-bonding, despite the oxygen atoms being potential acceptors.

### 2.3. X-Ray Powder Diffraction

X-ray powder diffractograms of the bulk powder precipitates showed four distinct phases, consistent with the simulated diffractograms obtained from structural data of crystalline Ce (**3Ce**), Y (**6Y**), Dy (**5Dy**), and Er (**7Er**) complexes. Diffractograms of the starting materials and co-products (NaCl, NaNO_3_, RECl_3_, RE(NO_3_)_3_ and 4npaH) were compared to the bulk precipitate powder diffractograms for each complex and no such impurities were detected.

**Figure 7:**
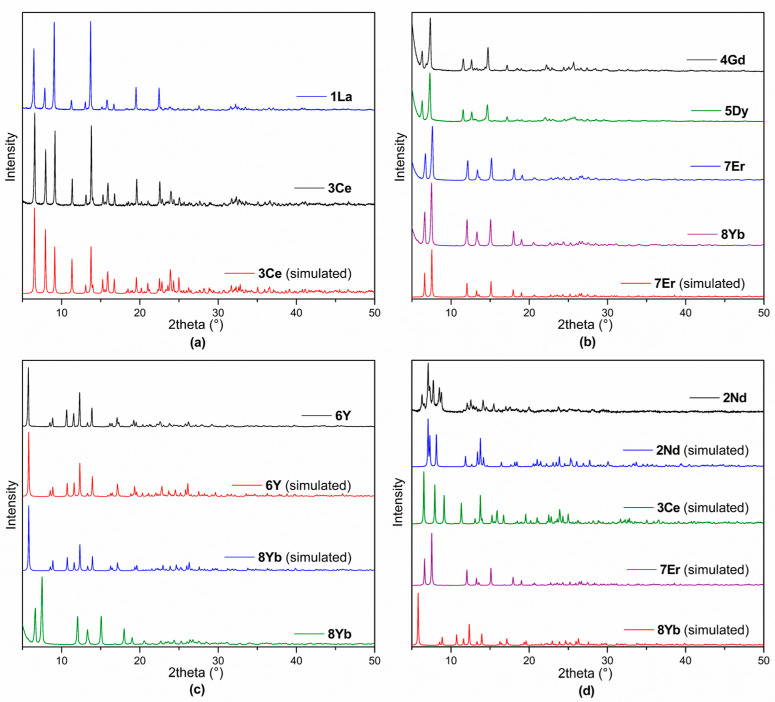
XRPD traces for La (**1La**), Nd (**2Nd**), Ce (**3Ce**), Gd (**4Gd**), Dy (**5Dy**), Y (**6Y**), Er (**7Er**) and Yb (**8Yb**): (**a**) represents powder precipitates isostructural with the crystalline Ce (**3Ce**) complex; (**b**) represents powder precipitates isostructural with the crystalline Er (**7Er**) complex; (**c**) represents powder precipitates isomorphous with the crystalline Yb (**8Yb**) complex with exception of Yb (**8Yb**) XRPD; and (**d**) shows Nd (**2Nd**) XRPD, which does not correspond to any generated from crystal structures.

The simulated XRPDs and bulk powder XRPDs of the Ce (**3Ce**), Y (**6Y**), Dy (**5Dy**), and Er (**7Er**) complexes match (Figure 7). The powder diffractogram of the La complex **1La** corresponds to that of Ce complex **3Ce**, indicating the loss of water of crystallization on isolation of the powder or on grinding for powder measurement. The Nd (**2Nd**) powder diffractogram does not resemble any simulated trace (Figure 7d), suggesting partial dehydration, which is supported by TGA data indicating that the powder has only one water (Figure S11). The Gd (**4Gd**) XRPD and Yb (**8Yb**) XRPD match the Er (**7Er**) SCXRD. Thus, the Gd complex is isomorphous with **5Dy** and **7Er**. The result for the Yb complex indicates that the structure of the powder differs from that of the single crystals. (The single crystal form was maintained after variations in crystallization.) Accordingly, complex **8Yb** exhibits coordination isomerism (CN of Yb2 = 8 in single crystals and 9 in powder.) A more spectacular example is shown by dysprosium cinnamate, which has forms with nine and seven coordination [40].

The coordination isomerism of the Yb (**8Yb**) complex is a result of the bridging mode of the carboxylate O9,O10 in Figure 6 bridging Y1 and Y2, μ-1κ(O)-2κ(O′) in the Y (**6Y**) and hence in the isomorphous crystalline Yb (**8Yb**) complex, changing to μ-1κ(O)2κ(O,O′) in the powder form of **8Yb,** as is also observed in the crystalline Er (**7Er**) and Dy (**5Dy**) complexes. Thus, there is a coordination number discontinuity for RE2 observed in the sequence **5Dy** (CN9), **6Y** (CN8), **7Er** (CN9), **8Yb** (powder) (CN9), **8Yb** (crystals) (CN8).

### 2.4. Corrosion Inhibition

The corrosion inhibition values (Table 3) were obtained after immersion of AS1020 mild steel coupons in the specified inhibitor solutions for 168 h. The results indicated that the 4npa complexes inhibit corrosion in mild steel, with significantly less pitting observed for the coupons submerged in the test solutions relative to the control. Similar corrosion inhibition was observed for 4npa complexes relative to previous studies using 3-furoate, 4-hydroxycinnamate, and 2-thiophenecarboxylate, ligands that achieved up to 90% inhibition [13,41,42]. The most significant inhibition efficiency was 89%, observed for a 500 ppm solution of the {[Dy_2_(4npa)_6_(H_2_O)]·2H_2_O}_n_ bulk powder (Table 3). The 4npa complexes exhibit greater IE% than those of phenylacetate alone and 2-hydroxyphenyl acetate [43]. Furthermore, both 4-hydroxy and 4-nitro substituted phenylacetate have comparable performance in terms of corrosion inhibition in all cases except for the ineffective Ce (**3Ce**) complex (Table 3), which is not understood. Thus, this similarity between the effects of 4-hydroxy and 4-nitro substituents on corrosion inhibition is consistent with earlier results for similarly substituted cinnamate complexes [23], and it occurs despite the electronic dissimilarity in the substituents. However, both substituents have the capacity to coordinate to a steel surface. Despite the differences in the solid state structures, it may be expected that the polymers break down into monomeric species at the dilutions used for corrosion testing, consistent with prior observations in salicylate hydrate [44]. If 4-nitrophenylacetates were to be used industrially, then the rare earth costs might dictate a preference for well-performing Nd or Gd complexes over Dy.

## 3. Materials and Methods

### 3.1. General Consideration

The syntheses were conducted using commercially available chemicals obtained from Merck Life Science Pty Ltd. (Melbourne, VIC, Australia) and additional metal salt reagents of standard quality without undergoing any purification steps. Elemental analyses were performed by the Elemental Analysis Service Team, Science Centre, London Metropolitan University, London, England. Melting points were determined in glass capillaries, with the values reported without calibration. Infrared spectra were obtained with a Thermo-Scientific Nicolet™ iS™ 5 FT-IR spectrophotometer (Thermo Fisher Scientific, Waltham, MA, USA) fitted with an iD5 ATR attachment and a ZnSe crystal. Spectra were obtained in the range of 4000–400 cm^−1^ and processed with OMNIC 9.13.1256 software Powder XRD measurements were obtained at room temperature with a Bruker D2 PHASER diffractometer (Bruker corporation, Billerica, MA, USA) in the range of 5–50° with a 1° divergence slit and at 0.02° increments. X-ray powder simulations were generated by the Mercury program (Mercury 2024.3.1), provided by Cambridge Crystallographic Data Centre [45], from the obtained single-crystal X-ray diffraction data. TGA was performed with a TA instrument SDT 650 (TA Instruments, New Castle, DE, USA)using standard 90 µL alumina sample pans. The tests were programmed ranging from room temperature to 750 °C with a ramp of 10 °C per min.

### 3.2. X-Ray Crystallography

The structures of the complexes were determined by mounting single crystals on loops using viscous hydrocarbon oil and measuring the diffraction on either a Bruker D8 Quest (Bruker, Karlsruhe, Germany) using Mo-Kα radiation (λ = 0.71073 Å) at 293 K (**3Ce**-**8Yb**) or on the Australian Synchrotron MX1 beamline (Clayton, VIC, Australia) at 100 K for the smaller crystals (**1La**-**2Nd**). Structural solutions were determined using SHELXT intrinsic phasing method and refined using full-matrix least-squares methods against F2 using SHELX2015, [46], in conjunction with the Olex2, [47] graphical user interface. All the hydrogen atoms were placed in calculated positions using the riding model. Crystal data and refinement details are given in the Supplementary Information (Table S1). Deposition numbers, CCDC 2481978-2481980 for compounds **1La**-**3Ce** and 2481981-2481984 for compounds **5Dy**-**8Yb**, contain the supplementary crystallographic data for this paper. These data are provided free of charge by the Cambridge Crystallographic Data Centre via www.ccdc.cam.ac.uk/data_request/cif.

### 3.3. Synthesis of Rare Earth 4-Nitrophenylacetate Complexes

Aqueous sodium hydroxide (0.5 M, 3 mL) was added to 4npaH (1.5 mmol) dissolved in 10 mL of ethanol and water (1:4 *v*/*v*) by sonication, giving a solution of pH ca. 7. This solution was then slowly added to the rare earth hydrated nitrates or chlorides (0.5 mmol), with the former used for Y, La, Gd, Dy, and Er and the latter for Ce, Nd, and Yb complexes, in a minimal amount of water with the reaction mixture at pH ca. 6.

After 1 h, the resulting precipitate was filtered off and dried at ~50–60 °C before being left to stand in a desiccator for several days before being collected for analysis. The filtrate was transferred to a separate vial and allowed to evaporate slowly for days/weeks to facilitate crystallisation.

**1La**: {[La(4npa)_3_(H_2_O)_2_]·2H_2_O}_n_ Colourless crystals. Yield 82% (bulk powder). m.p. ~245 °C (dec). Solubility 1.512 g/L (1512 ppm, 2.01 mM). Elemental analysis for C_24_H_26_LaN_3_O_16_ (Mw: 751.39 g/mol^−1^); Calculated (%) C 38.36; H 3.49; N 5.59; La 18.49. Found (%) C 38.63; H 2.70; N 5.67; La 18.18. IR (*ν*/cm^−1^): 3629w, 3317wbr, 1624w, 1603w 1556s, 1510vs, 1408s, 1394s, 1341vs, 1276m, 1199w, 1156w, 1108w, 1015w, 939w, 879w, 856w, 822w, 771w, 719s, 670s 629w, 591m, 490m, 450m, 406w. TGA weight loss (25–95 °C); 9.1% (calc. for loss of 4 × H_2_O = 9.6%).

**2Nd**: {[Nd(4npa)_3_(H_2_O)_2_]·2H_2_O}_n_ Pale pink crystals. Yield 93% (bulk powder). m.p. ~240–244 °C (dec). Solubility 1.329 g/L (1329 ppm, 1.8 mM). Elemental analysis for C_24_H_26_N_3_NdO_16_ (Mw: 756.72 g/mol^−1^); Calculated (%) C 38.09; H 3.46; N 5.55; Nd 19.06. Found (%) C 38.61; H 2.63; N 5.63; Nd 18.75. IR (*ν*/cm^−1^): 3629w, 3588w, 3209wbr, 1600w, 1560m, 1508vs, 1425m, 1394m, 1343vs, 1277m, 1201w, 1181w, 1108w, 1017w, 944w, 874w, 856w, 820w, 762w, 715s, 678m, 664w, 630w, 578m, 493w, 451m, 409m. TGA weight loss (25–95 °C); 2.2% (calc. for loss of 1 × H_2_O = 2.4%).

**3Ce**: [Ce(4npa)_3_(H_2_O)_2_]_n_ Pale yellow crystals. Yield 86% (bulk powder). m.p. ~215–225 °C (dec). Solubility 3.191 g/L (3191 ppm, 4.45 mM). Elemental analysis for C_24_H_22_CeN_3_O_14_ (Mw: 716.56 g/mol^−1^); Calculated (%) C 40.23; H 3.09; N 5.86; Ce 19.56. Found (%) C 40.14; H 2.94; N 5.54; Ce 18.90. IR (*ν*/cm^−1^): 3630w, 3547w, 3316wbr, 1623w, 1602w, 1585w, 1552s, 1510vs, 1401s, 1340vs, 1312s, 1283s, 1200w, 1182w, 1159w, 1107m, 1014w, 945m, 877w, 857m, 821m, 769w, 726s, 662s, 630m, 585m, 525m, 487m, 441m. TGA weight loss (25–95 °C); 4.7% (calc. for loss of 2 × H_2_O = 5.0%).

**4Gd**: {[Gd_2_(4npa)_6_(H_2_O)]·2H_2_O}_n_ Yellow crystals. Yield 83% (bulk powder). m.p. ~230–232 °C (dec). Solubility 1.789 g/L (1789 ppm, 1.2 mM). Elemental analysis for C_48_H_42_Gd_2_N_6_O_27_ (Mw: 1449.37 g/mol^−1^); Calculated (%) C 39.78; H 2.92; N 5.80; Gd 21.70. Found (%) C 39.22; H 2.35; N 5.86; Gd 22.04. IR (*ν*/cm^−1^): 3588s, 3209s, 1545m, 1508vs, 1428w, 1399m, 1341vs, 1278w, 1201s, 1182s, 1108m, 1016s, 947m, 856m, 821w, 763m, 735m, 715w, 680w, 662w, 630m, 578w, 496w, 451w, 412w. TGA weight loss (25–95 °C); 4.1% (calc. for loss of 3 × H_2_O = 3.7%).

**5Dy**: {[Dy_2_(4npa)_6_(H_2_O)]·2H_2_O}_n_ Yellow crystals. Yield 70% (bulk powder). m.p. ~250–255 °C (dec). Solubility 1.415 g/L (1415 ppm, 1.0 mM). Elemental analysis for C_48_H_42_Dy_2_N_6_O_27_ (Mw: 1459.87 g/mol^−1^); Calculated (%) C 39.49; H 2.90; N 5.76; Dy 22.26. Found (%) C 39.10; H 2.28; N 5.66; Dy 21.37. IR (*ν*/cm^−1^): 3568s, 1624m, 1576m, 1508vs, 1429w, 1386m, 1339vs, 1274w, 1200m, 1181s, 1107m, 1016s, 947m, 853w, 820w, 761m, 736m, 716w, 681w, 664m, 630m, 577w, 493w, 454w. TGA weight loss (25–95 °C); 3.9% (calc. for loss of 3 × H_2_O = 3.7%).

**6Y**: {[Y_2_(4npa)_6_(H_2_O)]·2H_2_O}_n_ Yellow crystals. Yield 73% (bulk powder). m.p. *ca*. 255 °C (dec). Solubility 3.019 g/L (3019 ppm, 2.3 mM). Elemental analysis for C_48_H_42_N_6_O_27_Y_2_ (Mw: 1312.69 g/mol^−1^); Calculated (%) C 43.92; H 3.22; N 6.40; Y 13.55. Found (%) C 43.51; H 2.73; N 6.20; Y 12.97. IR (*ν*/cm^−1^): 3620w, 3115w, 1601w, 1541s, 1504vs, 1428s, 1393s, 1340vs, 1280s 1184w, 1108m, 1015w, 948w, 937w, 854m, 819m, 764m, 735m, 716s, 682m, 660s, 630m, 577m, 483m, 458m. TGA weight loss (25–95 °C); 5.2% (calc. for loss of 3 × H_2_O = 4.1%).

**7Er**: {[Er_2_(4npa)_6_(H_2_O)]·2H_2_O}_n_ Pale pink crystals. Yield 86% (bulk powder). m.p. ~250–252 °C (dec). Solubility 2.787 g/L (2787 ppm, 1.90 mM). Elemental analysis for C_48_H_42_Er_2_N_6_O_27_ (Mw: 1469.39 g/mol^−1^); Calculated (%) C 39.23; H 2.88; N 5.72; Er 22.76. Found (%) C 38.89; H 2.48; N 5.71; Er 22.67. IR (*ν*/cm^−1^): 3650w, 3629w, 3620w, 3504w, 1618w, 1601w, 1549s, 1508vs, 1427m, 1400s, 1339vs, 1319m, 1278m, 1200w, 1177w, 1107w, 1016w, 938w, 852w, 820w, 774w, 765w, 719s, 683m, 657m, 630w, 584w, 497m, 474w, 458w, 413w. TGA weight loss (25–95 °C); 3.2% (calc. for loss of 3 × H_2_O = 3.7%).

**8Yb**: {[Yb_2_(4npa)_6_(H_2_O)]·2H_2_O}_n_ Colourless crystals. Yield 78% (bulk powder). m.p. ~260–265 °C (dec). Solubility 2.480 g/L (2480 ppm, 1.67 mM). Elemental analysis for C_48_H_42_N_6_O_27_Yb_2_ (Mw: 1480.95 g/mol^−1^); Calculated (%) C 38.93; H 2.86; N 5.67; Yb 23.37. Found (%) C 38.93; H 2.29; N 5.44; Yb 23.44. IR (*ν*/cm^−1^): 3609w, 3115w, 1601w, 1542s, 1507vs, 1428s, 1401s, 1338vs, 1279s, 1200w, 1178w, 1108m, 1016w, 938w, 852m, 819m, 766w, 719s, 683m, 658m, 630m, 584m, 498m, 455m, 401w. TGA weight loss (25–95 °C); 3.9% (calc. for loss of 3 × H_2_O = 3.6%).

### 3.4. Immersion Testing

Corrosion inhibition was determined by the standard methods ASTM G31-72 and ASTM G1-03 [48,49]. For the immersion test, AS1020 mild steel coupons with dimensions of approximately 20 × 20 × 3 mm were used, and the experimental procedure was carried out as reported in the literature [13,41,42,50]. Trials were performed in duplicate to ensure reproducibility of the corrosion inhibiting effect with variation of < 16%. Table 3 provides a summary of the results.

## 4. Conclusions

Rare earth metal ions form four structural classes of complexes with the 4npa ligand, namely {[RE(4npa)_3_(H_2_O)_2_]·2H_2_O}_n_ (RE = La, Nd), [Ce(4npa)_3_(H_2_O)_2_]_n_, {[RE_2_(4npa)_6_(H_2_O)]·2H_2_O}_n_ (RE = Gd, Dy, Er), and the isostoichiometric {[RE_2_(4npa)_6_(H_2_O)]·2H_2_O}_n_ (RE = Y, Yb). The first two classes have 10-coordinate rare earth complexes, differing in the presence or absence of water of crystallisation, the third class has one 9-coordinate and one 8-coordinate metal, and the fourth class has two 8-coordinate metals. In some cases, the structures of precipitated powders differ from those of single crystals. Thus, the La complex is isomorphous with Ce, the Nd powder is partially dehydrated and, more interestingly, the Yb complex powder is isomorphous with the Dy and Er complexes and thus exhibits coordination isomerism. The single crystal structure sequence **5Dy**, **6Y**, **7Er** has a discontinuity in the coordination number–size correlation and thus differs from behaviour usually exhibited by lanthanoid carboxylates [1]. Corrosion inhibition tests show that mid-size rare earths Dy, Nd, and Gd complexes are very effective against the corrosion of mild steel, and that the 4-nitrophenylacetates are superior in performance to unsubstituted phenylacetates and 2-hydroxyphenylacetates and comparable to 4-hydroxyphenylacetates. Despite the electronic difference in the 4-substituent, both can potentially coordinate to a steel surface. Considering the cost of the rare earth metals, Nd or Gd 4npa compounds would be more industrially preferable to the Dy 4npa compound. Testing of the corrosion is handled over 7 days in aqueous NaCl solution, while crystallization of material from an aqueous solution can take several weeks. In all cases, we see no decomposition of the materials, suggesting they should be useful for applications in aqueous solution.

## Figures and Tables

**Table 1:** Selected infrared bands cm^−1^ of the RE 4-nitrophenylacetate complexes. ν(NO_2_) represents both asymmetric and symmetric stretching modes of the NO_2_ group, respectively.

Compound	ν(NO_2_)	ν_as_(COO^−^)	ν_s_(COO^−^)	Δν = (ν_as_ − ν_s_)
4npaH	1508, 1339	-	-	-
{[La(4npa)_3_(H_2_O)_2_]·2H_2_O}_n_ (**1La**)	1510, 1341	1556	1394	162
{[Nd(4npa)_3_(H_2_O)_2_]·2H_2_O}_n_ (**2Nd**)	1508, 1343	1560	1394	166
[Ce(4npa)_3_(H_2_O)_2_]_n_ (**3Ce**)	1510, 1340	1552	1401	151
{[Gd_2_(4npa)_6_(H_2_O)]·2H_2_O}_n_ (**4Gd**)	1508, 1341	1545	1399	146
{[Dy_2_(4npa)_6_(H_2_O)]·2H_2_O}_n_ (**5Dy**)	1508, 1339	1576	1386	190
{[Y_2_(4npa)_6_(H_2_O)]·2H_2_O}_n_ (**6Y**)	1504, 1340	1541	1393	148
{[Er_2_(4npa)_6_(H_2_O)]·2H_2_O}_n_ (**7Er**)	1508, 1338	1549	1400	149
{[Yb_2_(4npa)_6_(H_2_O)]·2H_2_O}_n_ (**8Yb**)	1507, 1338	1542	1401	141

**Table 2:** Average RE-O (carboxylate) bond lengths in RE 4-nitrophenylacetate complexes (CN = coordination number).

Compound	RE1		RE2	
	CN	RE1-O [Å]	CN	RE2-O [Å]
{[La(4npa)_3_(H_2_O)_2_]·2H_2_O}_n_ (**1La**)	10	2.59	-	-
{[Nd(4npa)_3_(H_2_O)_2_]·2H_2_O}_n_ (**2Nd**)	10	2.54	-	-
[Ce(4npa)_3_(H_2_O)_2_]_n_ (**3Ce**)	10	2.58	-	-
{[Dy_2_(4npa)_6_(H_2_O)]·2H_2_O}_n_ (**5Dy**)	8	2.36	9	2.41
{[Y_2_(4npa)_6_(H_2_O)]·2H_2_O}_n_ (**6Y**)	8	2.36	8	2.38
{[Er_2_(4npa)_6_(H_2_O)]·2H_2_O}_n_ (**7Er**)	8	2.36	9	2.41
{[Yb_2_(4npa)_6_(H_2_O)]·2H_2_O}_n_ (**8Yb**)	8	2.33	8	2.35

**Table 3:** Corrosion rates (mm year^−1^) and percentage inhibition efficiency (IE%) of compounds (**1La**-**8Yb**), 2hpa [43], 4hpa [24] and PhAc [25] for mild steel coupons immersed in inhibitor solutions of 500 ppm in 0.01 M NaCl after 7days (168 h) of immersion.

Solution	Concentration		Solubility	Ave. Weight Loss (mg)	Ave. Corrosion Rate (mm year^−1^)	IE%
	(ppm)	(mM)	In H_2_O (ppm)			
Control (NaCl)	500	0.01		14.2	0.101	-
{[La(4npa)_3_(H_2_O)_2_]·2H_2_O}_n_ (**1La**)	500	0.666	1512	4.5	0.030	**71**
{[Nd(4npa)_3_(H_2_O)_2_]·2H_2_O}_n_ (**2Nd**)	500	0.674	1329	2.1	0.014	**86**
{[Nd_2_(2hpa)_6_(H_2_O)_4_]⋅3.5H_2_O}_n_	500	0.378	-	-	0.051	48
{[Nd_2_(4hpa)_6_(H_2_O)]·4H_2_O}_n_	500	0.389	-	-	0.031	77
[Nd(PhAc)_3_(H_2_O)]_n_	500	-	-	13.6	0.449	55
[Ce(4npa)_3_(H_2_O)_2_]_n_ (**3Ce**)	500	0.708	3191	10.9	0.072	**29**
{[Ce(2hpa)_6_(H_2_O)_4_]⋅3.5H_2_O}_n_	500	0.380	-	-	0.074	24
{[Ce(4hpa)_3_(H_2_O)_2_]·H_2_O}_n_	500	0.772	-	-	0.043	68
{[Gd_2_(4npa)_6_(H_2_O)]·2H_2_O}_n_ (**4Gd**)	500	0.347	1789	3.0	0.020	**80**
{[Gd_2_(2hpa)_6_(H_2_O)_2_]⋅3H_2_O}_n_	500	0.381	-	-	0.0430	57
{[Gd_2_(4hpa)_6_(H_2_O)]·4H_2_O}_n_	500	0.381	-	-	0.0160	88
[Gd(PhAc)_3_(H_2_O)]_n_	500	-	-	16.4	0.5430	45
{[Dy_2_(4npa)_6_(H_2_O)]·2H_2_O}_n_ (**5Dy**)	500	0.351	1415	1.8	0.0116	**89**
{[Dy_2_(2hpa)_6_(H_2_O)_2_]⋅3H_2_O}_n_	500	0.378	-	-	0.0590	40
{[Dy_2_(4hpa)_6_(H_2_O)]·3H_2_O}_n_	500	0.383	-	-	0.0360	73
[Dy(PhAc)_3_(H_2_O)]_n_	500	-	-	11.0	0.3640	21
{[Y_2_(4npa)_6_(H_2_O)]·2H_2_O}_n_ (**6Y**)	500	0.382	3019	3.7	0.0247	**76**
{[Er_2_(4npa)_6_(H_2_O)]·2H_2_O}_n_ (**7Er**)	500	0.345	2787	3.8	0.0253	**75**
{[Yb_2_(4npa)_6_(H_2_O)]·2H_2_O}_n_ (**8Yb**)	500	0.340	2480	4.7	0.0313	**69**

## Data Availability

The data that support the findings of this study are available from the corresponding author upon reasonable request.

## Supplementary Materials

The following supporting information can be downloaded at https://www.mdpi.com/article/10.3390/molecules30193940/s1, Figure S1: Stacked plots of ATR-FTIR spectra of 4npaH (top) followed sequentially by complexes (**1La**-**8Yb**); {[RE(4npa)_3_(H2O)_2_]·2H_2_O}_n_ (RE = La (**1La**), Nd (**2Nd**)), [Ce(4npa)_3_(H_2_O)_2_]_n_ (**3Ce**), {[RE_2_(4npa)_6_(H_2_O)]·2H_2_O}_n_ (RE = Gd (**4Gd**), Dy (**5Dy**), Y (**6Y**), Er (**7Er**), Yb (**8Yb**)), (4npa = 4-nitrophenylacetate); Figure S2: ATR-FTIR spectrum of the starting material 4npaH; Figure S3: ATR-FTIR spectrum of {[La(4npa)_3_(H_2_O)_2_]·2H_2_O}_n_ (**1La**); Figure S4: ATR-FTIR spectrum of {[Nd(4npa)_3_(H_2_O)_2_]·2H_2_O}_n_ (**2Nd**); Figure S5: ATR-FTIR spectrum of [Ce(4npa)_3_(H_2_O)_2_]_n_ (**3Ce**); Figure S6: ATR-FTIR spectrum of {[Gd_2_(4npa)_6_(H_2_O)]·2H_2_O}_n_ (**4Gd**); Figure S7: ATR-FTIR spectrum of {[Dy_2_(4npa)_6_(H_2_O)]·2H_2_O}_n_ (**5Dy**); Figure S8: ATR-FTIR spectrum of {[Y_2_(4npa)_6_(H_2_O)]·2H_2_O}_n_ (**6Y**); Figure S9: ATR-FTIR spectrum of {[Er_2_(4npa)_6_(H_2_O)]·2H_2_O}_n_ (**7Er**); Figure S10: ATR-FTIR spectrum of {[Yb_2_(4npa)_6_(H_2_O)]·2H_2_O}_n_ (**8Yb**); Figure S11: TGA plots of {[RE(4npa)_3_(H_2_O)_2_]·2H_2_O}_n_ (RE = La (**1La**), Nd (**2Nd**)), [Ce(4npa)_3_(H_2_O)_2_]_n_ (**3Ce**), {[RE_2_(4npa)_6_(H_2_O)]·2H_2_O}_n_ (RE = Gd (**4Gd**), Dy (**5Dy**), Y (**6Y**), Er (**7Er**), Yb (**8Yb**)), (4npa = 4-nitrophenylacetate); Table S1: Crystal data and structural refinement for RE 4npa complexes; Table S2: Selected bond lengths, Ln…Ln distances (Å) and selected bond angles (°) for the second structural type {[RE(4npa)_3_(H_2_O)_2_]·2H_2_O}_n_ (RE = La, Nd) complexes **1La** and **2Nd**; Table S3: Hydrogen bonds for {[RE(4npa)_3_(H_2_O)_2_]·2H_2_O}_n_ (RE = La, Nd) complexes **1La** and **2Nd** [d/Å and </°]; Table S4: Selected bond lengths, La…La distances (Å) and selected bond angles (°) for the first structural type [Ce(4npa)_3_(H_2_O)_2_]_n_ complex **3Ce**; Table S5: Hydrogen bonds for [Ce(4npa)_3_(H_2_O)_2_]_n_ complex **3Ce** [d/Å and </°]; Table S6: Selected bond lengths, Ln…Ln distances (Å) and selected bond angles (°) for the third structural type {[RE_2_(4npa)_6_(H_2_O)]·2H_2_O}_n_ (RE = Dy, Er) complexes **5Dy** and **7Er**; Table S7: Hydrogen bonds for {[RE_2_(4npa)_6_(H_2_O)]·2H_2_O}_n_ (RE = Dy, Er) complexes **5Dy** and **7Er** [d/Å and </°]; Table S8: Selected bond lengths, Ln…Ln distances (Å) and selected bond angles (°) for the third structural type {[RE_2_(4npa)_6_(H_2_O)]·2H_2_O}_n_ (RE = Y, Yb) complexes **6Y** and **8Yb**; Table S9: Hydrogen bonds for {[RE_2_(4npa)_6_(H_2_O)]·2H_2_O}_n_ (RE = Y, Yb) complexes **6Y** and **8Yb** [d/Å and </°]; Table S10: TGA weight loss percentages around 110–265 or 300 °C for compounds **1**–**8** [47,51].
