# Supplementary material for: Synthesis, Structures and Corrosion Inhibition Properties of 4-Nitrophenylacetato-Rare-Earth(III) 1D Coordination Polymers
Source: Molecules. 2025 Oct 1;30(19):3940. doi: 10.3390/molecules30193940 (PMC12526171; doi:10.3390/molecules30193940)
Supplement: Supplementary file 1 [file molecules-30-03940-s001.zip › molecules-3879970-supplementary.pdf]

Supplementary Material

Synthesis, Structures and Corrosion Inhibition Properties of 4-Nitrophenylacetato-Rare-Earth(III) 1D Coordination Polymers

Jacob M. Neill [1], Naveena Y. Salpadoru Thuppahige [1], Zhifang Guo [1], Glen B. Deacon [2] and Peter C. Junk [1,*]

[1] College of Science & Engineering, James Cook University, Townsville, QLD 4811, Australia; jacob.neill1@my.jcu.edu.au (J.M.N.); naveena.salpadoruthuppahige@my.jcu.edu.au (N.Y.S.T.); zhifang.guo@jcu.edu.au (Z.G.)
[2] School of Chemistry, Monash University, Clayton, VIC 3800, Australia; glen.deacon@monash.edu
* Correspondence: peter.junk@jcu.edu.au

Contents:

Table S1. Crystal data and structural refinement for RE 4-nitrophenylacetate (RE-4npa) complexes.
Table S2. Selected bond lengths, Ln…Ln distances (Å) and Selected bond angles (°) for the second structural type $\{[RE(4npa)_3(H_2O)_2]\cdot 2H_2O\}_n$ (RE = La, Nd) complexes **1La** and **2Nd**.
Table S3. Hydrogen bonds for $\{[RE(4npa)_3(H_2O)_2]\cdot 2H_2O\}_n$ (RE = La, Nd) complexes **1La** and **2Nd** [d/Å and </°].
Table S4. Selected bond lengths, Ln…Ln distances (Å) and Selected bond angles (°) for the first structural type $[Ce(4npa)_3(H_2O)_2]_n$ complex **3Ce**.
Table S5. Hydrogen bonds for $[Ce(4npa)_3(H_2O)_2]_n$ complex **(3Ce)** [d/Å and </°].
Table S6. Selected bond lengths, Ln…Ln distances (Å) and Selected bond angles (°) for the third structural type $\{[RE_2(4npa)_6(H_2O)]\cdot 2H_2O\}_n$ (RE = Dy, Er) complexes **5Dy** and **7Er**.
Table S7. Hydrogen bonds for $\{[RE_2(4npa)_6(H_2O)]\cdot 2H_2O\}_n$ (RE = Dy, Er) complexes **5Dy** and **7Er** [d/Å and </°].
Table S8. Selected bond lengths, Ln…Ln distances (Å) and Selected bond angles (°) for the third structural type $\{[RE_2(4npa)_6(H_2O)]\cdot 2H_2O\}_n$ (RE = Y, Yb) complexes **6Y** and **8Yb**.
Table S9. Hydrogen bonds for $\{[RE_2(4npa)_6(H_2O)]\cdot 2H_2O\}_n$ (RE = Y, Yb) complexes **6Y** and **8Yb** [d/Å and </°].
Table S10. TGA weight loss percentages around 110 – 265 or 300 °C for compounds **1-8**.

Figure S1. Stacked plots of ATR-FTIR spectra of 4npaH (top) followed sequentially by complexes (**1La-8Yb**); $\{[RE(4npa)_3(H_2O)_2]\cdot 2H_2O\}_n$ (RE = La (**1La**), Nd (**2Nd**)), $[Ce(4npa)_3(H_2O)_2]_n$ (**3Ce**), $\{[RE_2(4npa)_6(H_2O)]\cdot 2H_2O\}_n$ (RE = Gd (**4Gd**), Dy (**5Dy**), Y (**6Y**), Er (**7Er**), Yb (**8Yb**)), (4npa = 4-nitrophenylacetate).
Figure S2. ATR-FTIR spectrum of the starting material 4npaH.
Figure S3. ATR-FTIR spectrum of $\{[La(4npa)_3(H_2O)_2]\cdot 2H_2O\}_n$ (**1La**).
Figure S4. ATR-FTIR spectrum of $\{[Nd(4npa)_3(H_2O)_2]\cdot 2H_2O\}_n$ (**2Nd**).
Figure S5. ATR-FTIR spectrum of $[Ce(4npa)_3(H_2O)_2]_n$ (**3Ce**).
Figure S6. ATR-FTIR spectrum of $\{[Gd_2(4npa)_6(H_2O)]\cdot 2H_2O\}_n$ (**4Gd**).

Figure S7. ATR-FTIR spectrum of $\{[Dy_2(4npa)_6(H_2O)]\cdot 2H_2O\}_n$ (**5Dy**).
Figure S8. ATR-FTIR spectrum of $\{[Y_2(4npa)_6(H_2O)]\cdot 2H_2O\}_n$ (**6Y**).
Figure S9. ATR-FTIR spectrum of $\{[Er_2(4npa)_6(H_2O)]\cdot 2H_2O\}_n$ (**7Er**).
Figure S10. ATR-FTIR spectrum of $\{[Yb_2(4npa)_6(H_2O)]\cdot 2H_2O\}_n$ (**8Yb**).
Figure S11. TGA plots of $\{[RE(4npa)_3(H_2O)_2]\cdot 2H_2O\}_n$ (RE = La (**1La**), Nd (**2Nd**)), $[Ce(4npa)_3(H_2O)_2]_n$ (**3Ce**), $\{[RE_2(4npa)_6(H_2O)]\cdot 2H_2O\}_n$ (RE = Gd (**4Gd**), Dy (**5Dy**), Y (**6Y**), Er (**7Er**), Yb (**8Yb**)), (4npa = 4-nitrophenylacetate). (**1La-8Yb** = La (**1**) – Yb (**8**) in the figure).

X-ray Crystallography

Complexes were measured on a Bruker D8 Quest using Mo-Kα radiation (λ = 0.71073 Å) at 293 K. Structural solutions were obtained by SHELXT intrinsic phasing method and refined using full-matrix least-squares methods against F2 using SHELX2015 [1], in conjunction with Olex2 [2] graphical user interface. All hydrogen atoms were placed in calculated positions using the riding model.

Table S1. Crystal data and structural refinement for RE 4-nitrophenylacetate (RE-4npa) complexes.

	La	Nd	Ce	Dy	Y	Er	Yb
**Formula**	$C_{24}H_{26}LaN_3O_{16}$	$C_{24}H_{26}N_3NdO_{16}$	$C_{24}H_{22}CeN_3O_{14}$	$C_{48}H_{42}Dy_2N_6O_{27}$	$C_{48}H_{42}N_6O_{27}Y_2$	$C_{48}H_{42}Er_2N_6O_{27}$	$C_{48}H_{42}N_6O_{27}Yb_2$
**M_r**	751.39	756.72	716.56	1459.87	1312.69	1469.39	1480.95
**Crystal System**	Triclinic	Triclinic	Triclinic	monoclinic	Monoclinic	Monoclinic	Monoclinic
**Space group**	P-1	P-1	P-1	$P2_1$	Cc	$P2_1$	Cc
**a (å)**	8.6900(17)	8.6000(17)	8.6384(3)	7.9317(6)	16.7421(12)	7.9043(6)	16.7411(6)
**b (å)**	13.250(3)	13.210(3)	11.5704(4)	12.9027(14)	39.836(3)	13.0432(14)	39.8165(16)
**c (å)**	13.430(3)	13.430(3)	14.1963(5)	26.396(2)	8.1707(5)	26.711(2)	8.1191(3)
**α (°)**	68.50(3)	68.54(3)	75.8400(10)	90	90	90	90
**β (°)**	86.06(3)	85.97(3)	77.5080(10)	92.511(3)	99.624(2)	92.360(3)	99.920(2)
**γ (°)**	79.09(3)	79.16(3)	80.2900(10)	90	90	90	90
**V (å^3)**	1412.8(6)	1394.6(6)	1333.16(8)	2698.8(4)	5372.7(6)	2751.5(4)	5331.1(3)
**Z**	2	2	2	2	4	2	4
**ρ_{calc}, g cm^{-3}**	1.766	1.802	1.785	1.797	1.623	1.774	1.845

**μ, mm^{-1}**	1.596	1.946	1.786	2.844	2.246	3.124	3.585
**N_τ**	33750	32578	35754	71105	74131	29371	83199
**N (R_{int})**	5324 (0.0238)	5237 (0.0486)	6098 (0.0680)	12576 (0.0645)	11857 (0.0653)	11437 (0.0786)	12112 (0.0894)
**R_1 (I > 2σ(I))**	0.0174	0.0416	0.0360	0.0356	0.0359	0.0468	0.0341
**wR_2 (all data)**	0.0451	0.1117	0.0870	0.0757	0.0808	0.0715	0.0749
**GOF**	1.052	1.079	1.066	1.023	0.981	1.013	1.037

Table S2. Selected bond lengths, Ln…Ln distances (Å) and Selected bond angles (°) for the second structural type {[RE(4npa)$_3$(H_2O)$_2$]·2H_2O}$_n$ (RE = La, Nd) complexes **1La** and **2Nd**.

	La1 (**1La**)	Nd1 (**2Nd**)
RE1*	4.3197(9)	4.2729(9)
RE1$^\#$	4.4081(9)	4.3667(9)
O5	2.4924(8)	2.4344(8)
O5$^\#$	2.6938(10)	2.6761(9)
O10*	2.5591(8)	2.5054(8)
O1	2.5871(8)	2.5260(8)
O2	2.6100(6)	2.5632(6)
O9*	2.6900(8)	2.6452(8)
O9	2.4931(8)	2.4451(8)
O6$^\#$	2.6135(12)	2.5500(11)
O13	2.6003(9)	2.5489(9)
O14	2.5895(6)	2.5184(6)

Bond angles	(**1La**)	(**2Nd**)
RE1$^\#$-RE1-RE1*	169.330(3)	169.020(4)
RE1-O5-RE1$^\#$	116.36(2)	117.32(2)
RE1-O5$^\#$-RE1$^\#$	116.36(2)	114.11(11)
RE1-O9-RE1*	112.850(19)	114.101(19)
RE1-O9*-RE1*	112.850(19)	114.101(19)

Table S3. Hydrogen bonds for $\{[RE(4npa)_3(H_2O)_2]\cdot 2H_2O\}_n$ (RE = La, Nd) complexes **1La** and **2Nd** [d/Å and </°].

D-H…A	d(D-H)	d(H…A)	d(D…A)	<(DHA)
**Complex 1La**				
O15-H15A…O2	0.8500(2)	1.878(5)	2.7237(7)	172.942(3)
O14*-H14*B…O15	0.8521(3)	1.8877(6)	2.7374(9)	174.816(3)
O13*-H13*A…O16	0.8570(2)	2.0212(9)	2.7209(12)	138.24(2)
O16-H16A…O6$^{\#}$	0.8499(2)	1.9263(6)	2.7501(8)	162.904(10)
O14-H14A…O1$^{\#}$	0.8525(3)	1.8625(5)	2.6949(7)	165.0(14)

Table S4. Selected bond lengths, Ln…Ln distances (Å) and Selected bond angles (°) for the first structural type $[Ce(4npa)_3(H_2O)_2]_n$ complex **3Ce**.

	Ce1 **(3Ce)**
Ce1*	4.32248(15)
Ce1$^{\#}$	4.38277(15)
O1	2.43231(6)
O5	2.57343(6)
O6	2.56302(8)
O9	2.45223(7)
O13	2.62747(7)
O14	2.63829(8)
O9*	2.69219(8)
O10*	2.57034(9)
O1$^{\#}$	2.75646(8)
O2$^{\#}$	2.56551(7)

Bond angles	**(3Ce)**
Ce1$^{\#}$-Ce1-Ce1*	165.7896(6)
Ce1-O1-Ce1$^{\#}$	115.130(2)
Ce1-O1$^{\#}$-Ce1$^{\#}$	115.130(2)
Ce1-O9-Ce1*	114.248(3)
Ce1-O9*-Ce1*	114.248(3)

Table S5. Hydrogen bonds for $[Ce(4npa)_3(H_2O)_2]_n$ complex **(3Ce)** [d/Å and </°].

D-H…A	d(D-H)	d(H…A)	d(D…A)	<(DHA)
O14-H14A…O6*	0.85131(3)	1.89229(6)	2.73525(9)	170.3368(4)
O13-H13A…O5$^{\#}$	0.85502(2)	1.93930(6)	2.77062(8)	163.7841(4)

Table S6. Selected bond lengths, Ln…Ln distances (Å) and Selected bond angles (°) for the third structural type $\{[RE_2(4npa)_6(H_2O)]\cdot 2H_2O\}_n$ (RE = Dy, Er) complexes **5Dy** and **7Er**.

	Dy1 (**5Dy**)	Er1 (**7Er**)
RE2	3.9866(3)	3.9767(3)
RE2$^{\#}$	3.9940(3)	3.9763(3)
O1	2.32755(13)	2.31051(13)
O5	2.24633(12)	2.25528(12)
O9	2.44624(16)	2.45140(15)
O10	2.41424(14)	2.41049(14)
O13	2.24313(17)	2.23180(17)
O17	2.41746(15)	2.42417(15)
O18	2.4654(2)	2.4767(2)
O21	2.31892(14)	2.30836(14)
	Dy2 (**5Dy**)	Er2 (**7Er**)
RE1*	3.9940(3)	3.9763(3)
O1*	2.55404(15)	2.55105(5)
O2*	2.39685(17)	2.39917(17)
O6*	2.29161(13)	2.29074(13)
O17*	2.37329(15)	2.36089(14)
O10	2.36743(15)	2.35117(15)
O14	2.3056(2)	2.2975(2)
O21	2.59012(16)	2.61648(16)
O22	2.36566(14)	2.38528(14)

O25	2.3783(2)	2.3788(2)

Bond angles	(**5Dy**)	(**7Er**)
RE2-RE1-RE2#	167.3129(15)	167.3107(15)
RE1-RE2-RE1*	167.3129(15)	167.3107(15)
RE1-O1-RE2#	109.718(5)	109.653(5)
RE1-O17-RE2#	112.955(6)	112.394(6)
RE1-O10-RE2	112.962(5)	113.257(5)
RE1-O21-RE2	108.475(13)	107.536(6)
RE2-O1*-RE1*	109.718(5)	109.653(5)
RE2-O17*-RE1*	112.955(6)	112.394(6)

Table S7. Hydrogen bonds for {[$RE_2(4npa)_6(H_2O)$]·$2H_2O$}$_n$ (RE = Dy, Er) complexes **5Dy** and **7Er** [d/Å and </°].

D-H…A	d(D-H)	d(H…A)	d(D…A)	<(DHA)
**Complex 5Dy**				
O25-H25B…O27	0.85489(5)	2.01212(12)	2.74235(17)	142.819(3)
O25#-H25A#…O26	0.84832(5)	1.83623(11)	2.66837(15)	166.4583(9)
**Complex 7Er**				
O25-H25B…O27	0.85342(5)	2.03973(13)	2.74857(17)	139.97(3)
O25#-H25A#…O26	0.85299(5)	1.81399(10)	2.65171(15)	166.8430(9)

Table S8. Selected bond lengths, Ln…Ln distances (Å) and Selected bond angles (°) for the third structural type {[$RE_2(4npa)_6(H_2O)$]·$2H_2O$}$_n$ (RE = Y, Yb) complexes **6Y** and **8Yb**.

	Y1 (**6Y**)	Yb1 (**8Yb**)
RE2	3.9850(2)	3.95024(14)
RE2*	4.2203(3)	4.20258(16)
O1	2.41430(12)	2.39100(6)
O2	2.48063(12)	2.45639(8)
O5	2.24703(11)	2.22238(6)
O9	2.25021(12)	2.21821(7)
O13	2.25463(11)	2.21450(6)

O17	2.45972(15)	2.44827(8)
O18	2.41301(11)	2.38417(7)
O21	2.36324(11)	2.33606(7)
	Y2 (**6Y**)	Yb2 (**8Yb**)
RE1$^{\#}$	4.2203(3)	4.20258(16)
O2$^{\#}$	2.38218(12)	2.36312(7)
O6$^{\#}$	2.30811(12)	2.27794(7)
O10$^{\#}$	2.29587(13)	2.25789(7)
O14	2.26516(12)	2.23576(6)
O18	2.40450(11)	2.38838(7)
O21	2.54747(13)	2.53289(8)
O22	2.42699(16)	2.39509(8)
O25	2.33185(17)	2.30002(9)

Bond angles	(**6Y**)	(**8Yb**)
RE2-RE1-RE2*	169.4739(9)	169.5699(5)
RE1-RE2-RE1$^{\#}$	169.4739(9)	169.5699(5)
RE1-O2-RE2*	120.410(4)	121.370(2)
RE1-O18-RE2	111.623(4)	111.726(2)
RE1-O21-RE2	108.426(5)	108.381(3)
RE2-O2$^{\#}$-RE1$^{\#}$	120.410(4)	121.370(2)

Table S9. Hydrogen bonds for {[RE_2(4npa)$_6$(H_2O)]·2H_2O}$_n$ (RE = Y, Yb) complexes **6Y** and **8Yb** [d/Å and </°].

D-H…A	d(D-H)	d(H…A)	d(D…A)	<(DHA)
**Complex 6Y**				
O25*-H25B*-O26	0.84986(4)	1.85427(9)	2.69507(13)	169.8932(9)

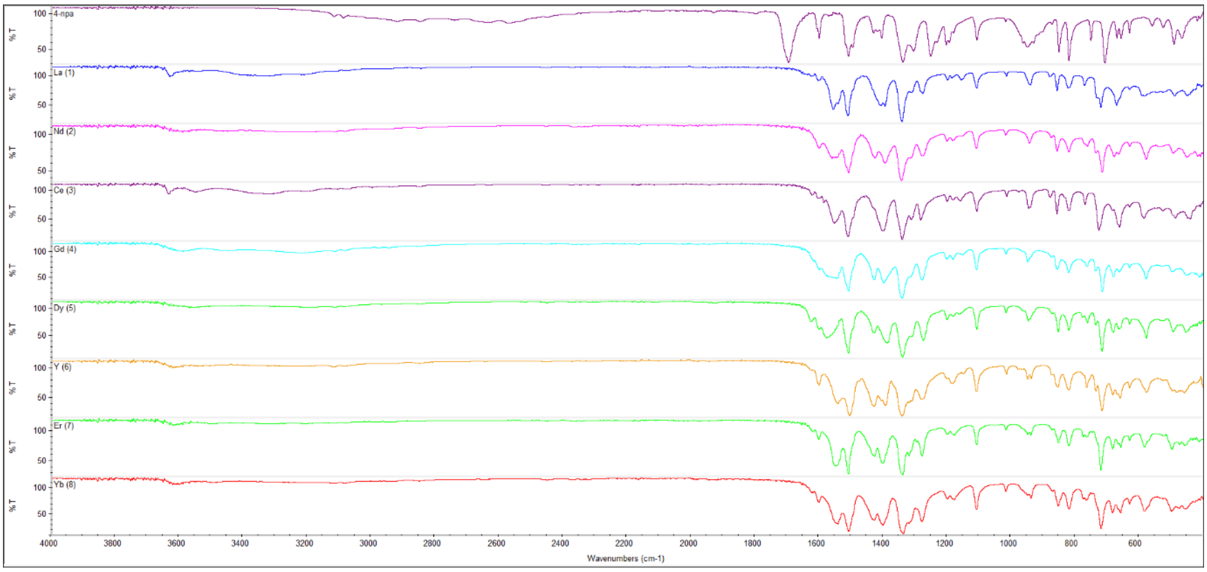

Figure S1. Stacked plots of ATR-FTIR spectra of 4npaH (top) followed sequentially by complexes (**1La-8Yb**); {[RE(4npa)$_3$(H_2O)$_2$]·2H_2O}$_n$ (RE = La (**1La**), Nd (**2Nd**)), [Ce(4npa)$_3$(H_2O)$_2$]$_n$ (**3Ce**), {[RE$_2$(4npa)$_6$(H_2O)]·2H_2O}$_n$ (RE = Gd (**4Gd**), Dy (**5Dy**), Y (**6Y**), Er (**7Er**), Yb (**8Yb**)), (4npa = 4-nitrophenylacetate).

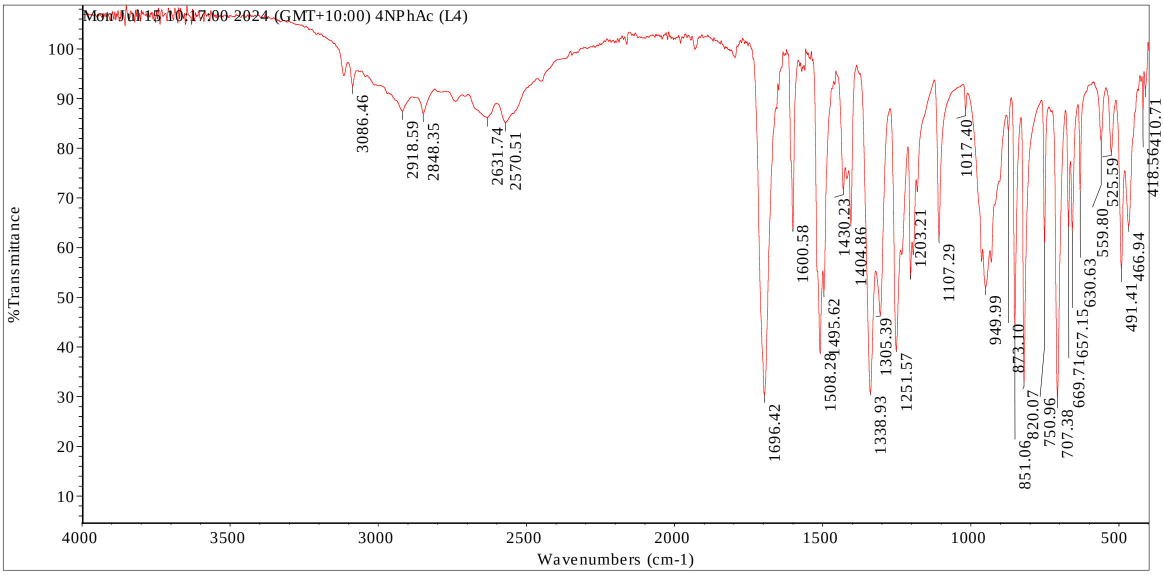

Figure S2. ATR-FTIR spectrum of the starting material 4npaH.

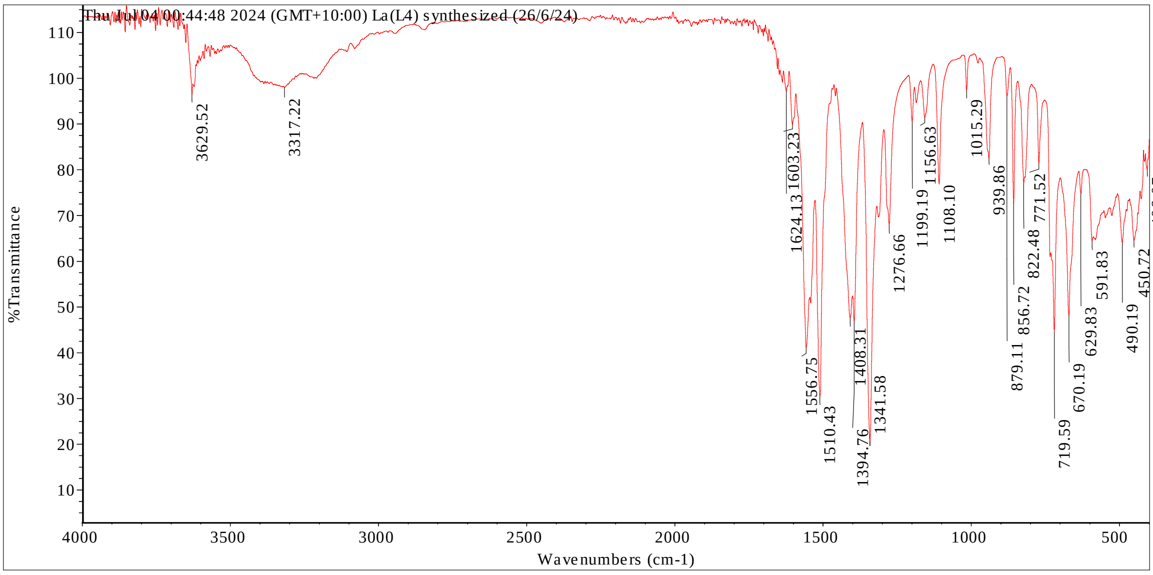

Figure S3. ATR-FTIR spectrum of $\{[La(4npa)_3(H_2O)_2]\cdot 2H_2O\}_n$ (**1La**).

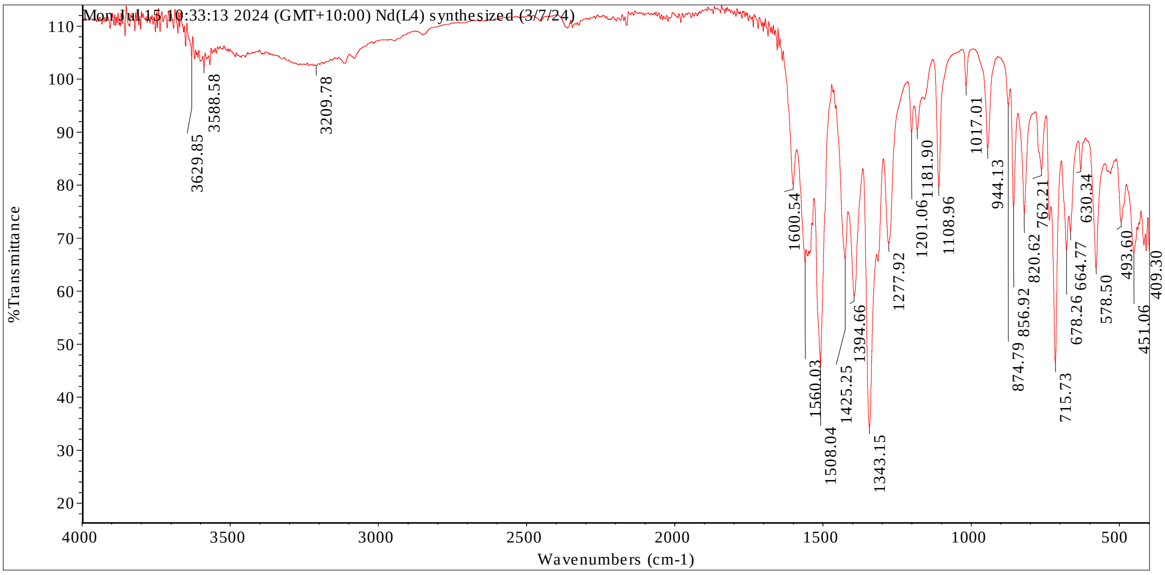

Figure S4. ATR-FTIR spectrum of $\{[Nd(4npa)_3(H_2O)_2]\cdot 2H_2O\}_n$ (**2Nd**).

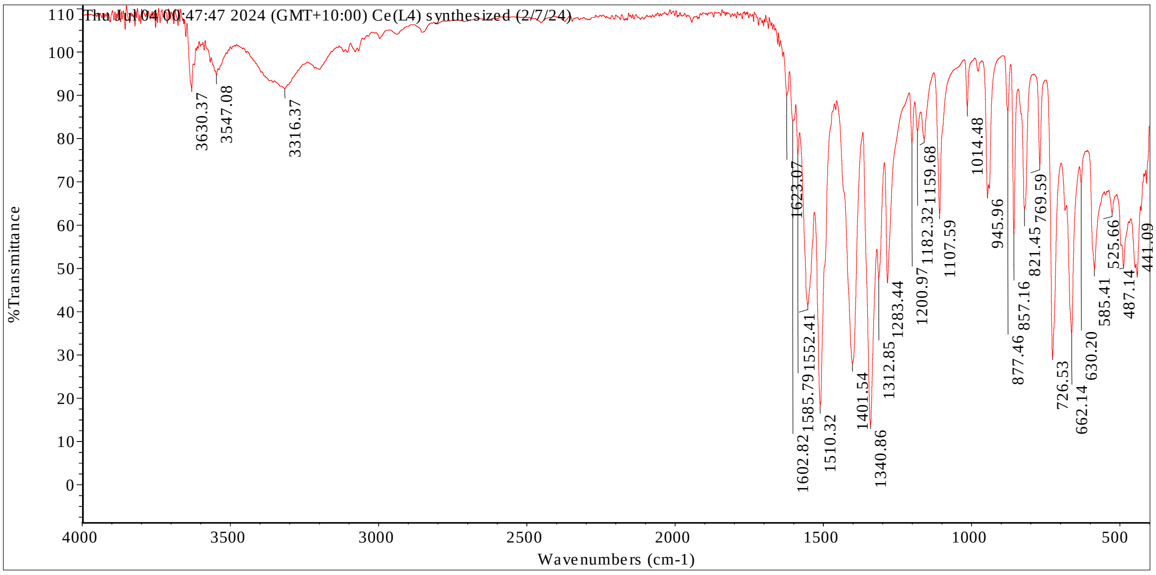

Figure S5. ATR-FTIR spectrum of $[Ce(4npa)_3(H_2O)_2]_n$ (**3Ce**).

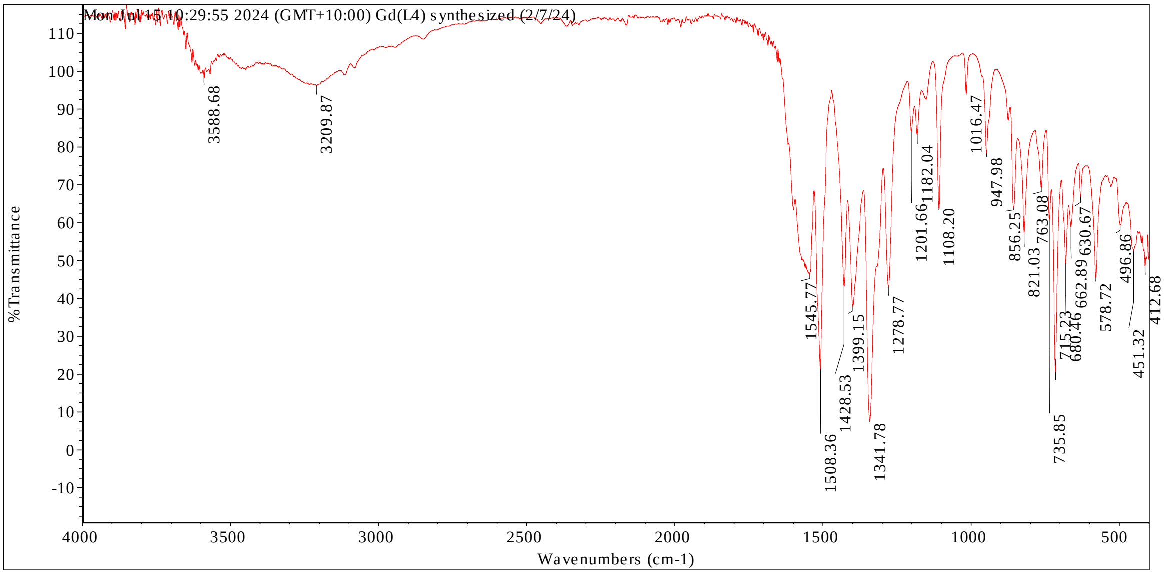

Figure S6. ATR-FTIR spectrum of $\{[Gd_2(4npa)_6(H_2O)]\cdot 2H_2O\}_n$ (**4Gd**).

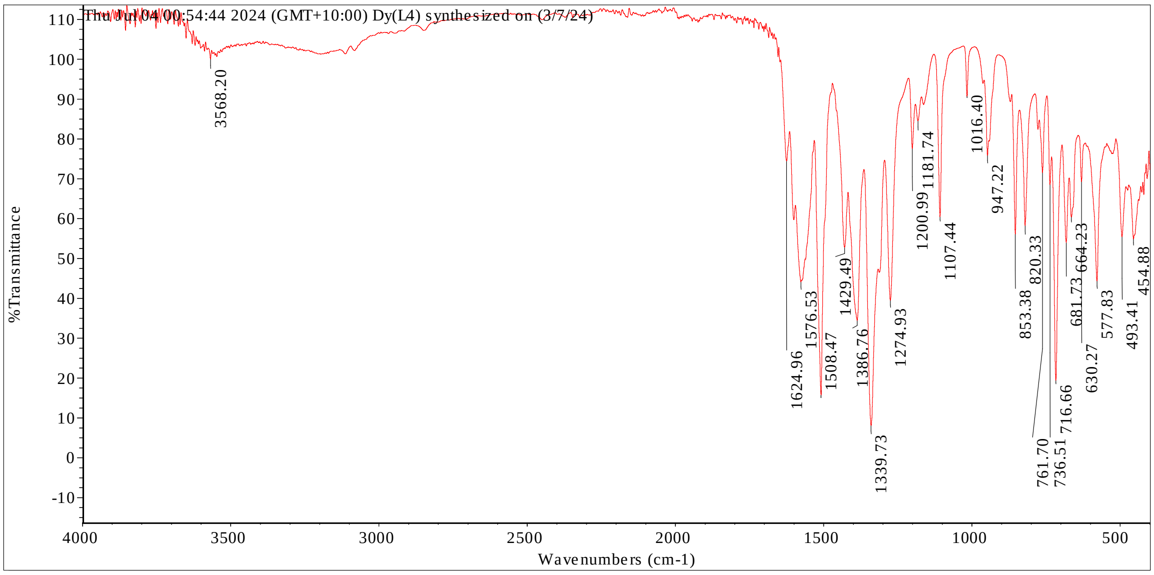

Figure S7. ATR-FTIR spectrum of $\{[Dy_2(4npa)_6(H_2O)]\cdot 2H_2O\}_n$ (**5Dy**).

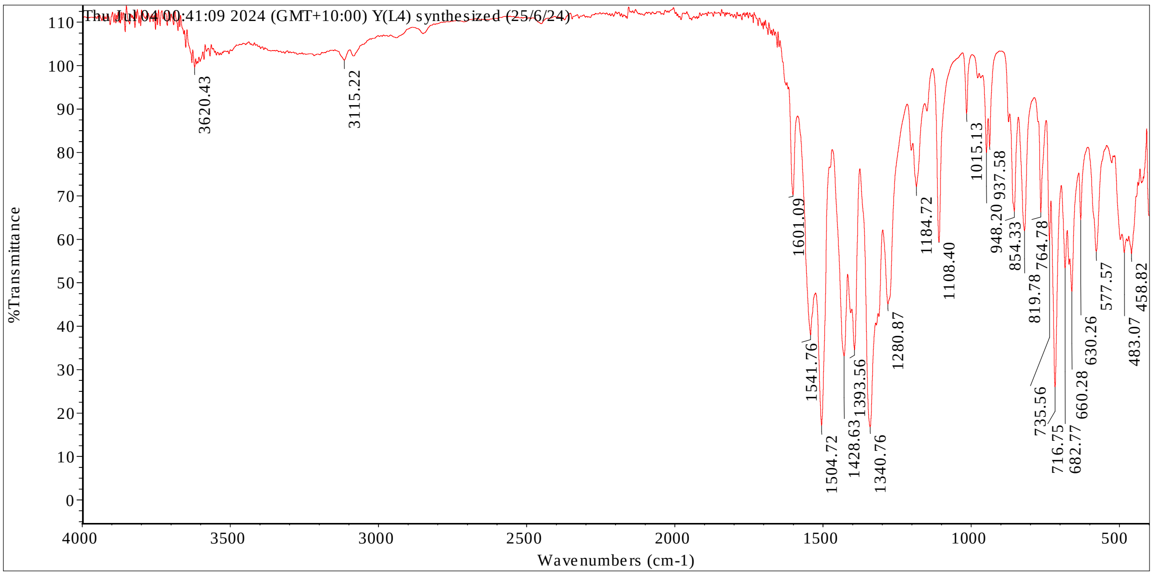

Figure S8. ATR-FTIR spectrum of $\{[Y_2(4npa)_6(H_2O)]\cdot 2H_2O\}_n$ (**6Y**).

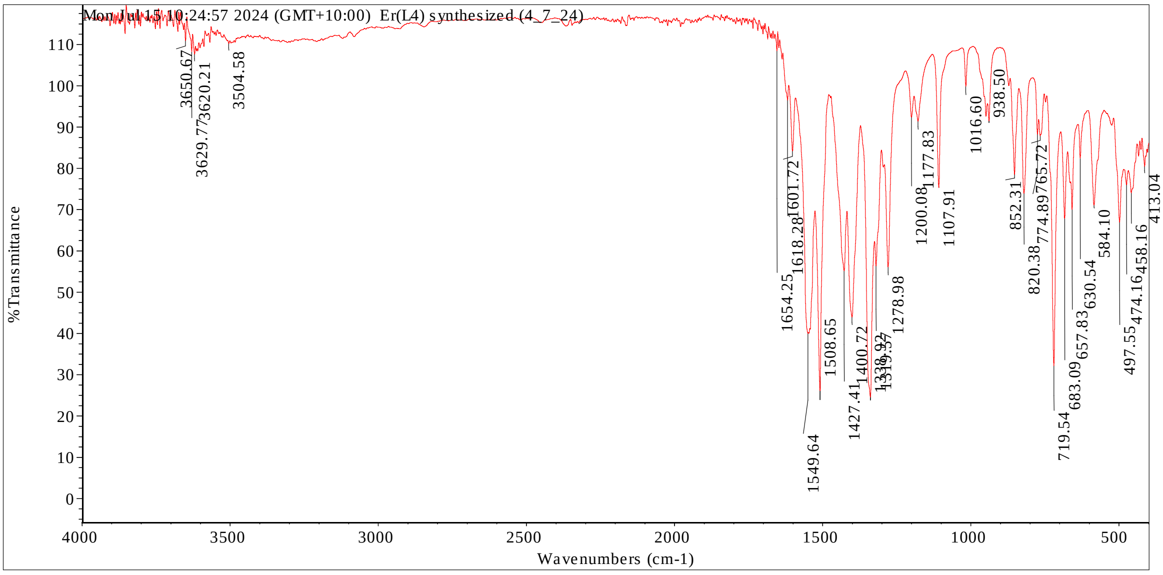

Figure S9. ATR-FTIR spectrum of $\{[Er_2(4npa)_6(H_2O)]\cdot 2H_2O\}_n$ (**7Er**).

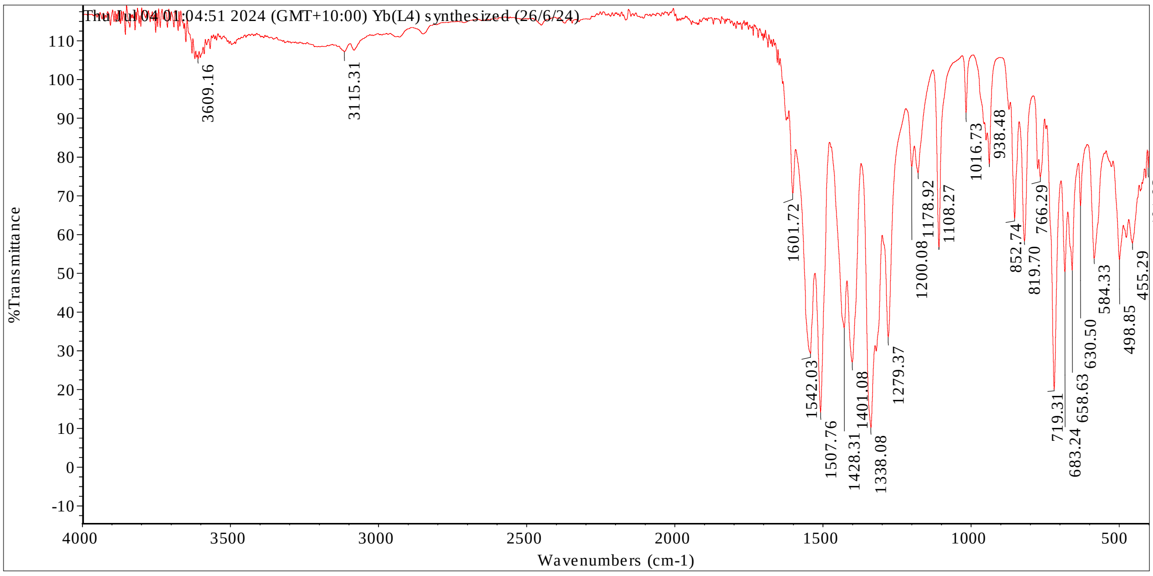

Figure S10. ATR-FTIR spectrum of $\{[Yb_2(4npa)_6(H_2O)]\cdot 2H_2O\}_n$ (**8Yb**).

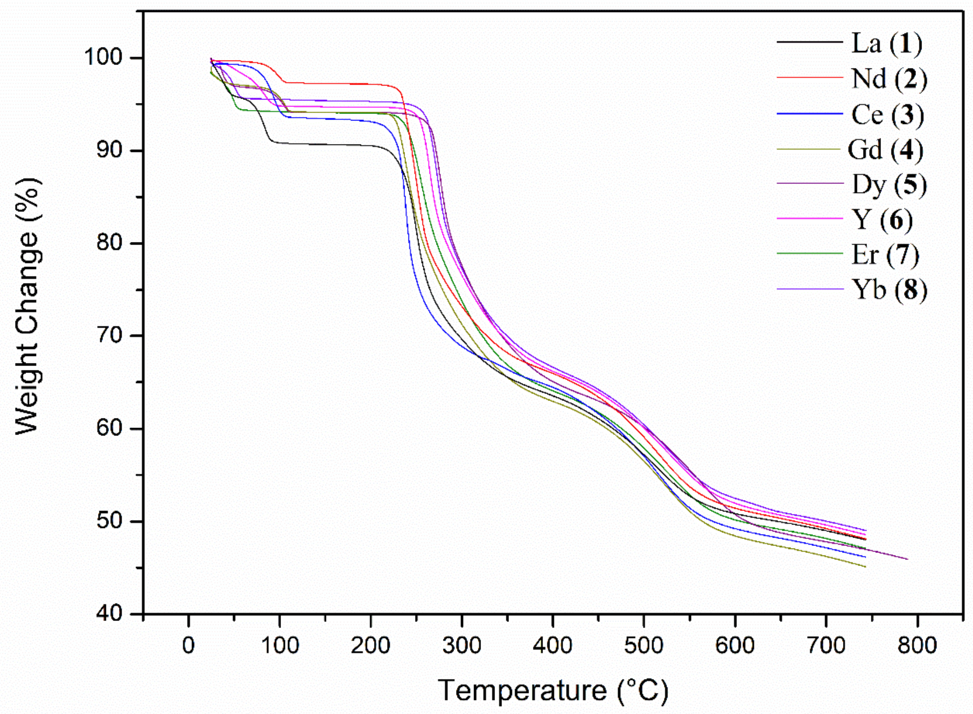

Figure S11. TGA plots of {[RE(4npa)$_3$(H$_2$O)$_2$]·2H$_2$O}$_n$ (RE = La (**1La**), Nd (**2Nd**)), [Ce(4npa)$_3$(H$_2$O)$_2$]$_n$ (**3Ce**), {[RE$_2$(4npa)$_6$(H$_2$O)]·2H$_2$O}$_n$ (RE = Gd (**4Gd**), Dy (**5Dy**), Y (**6Y**), Er (**7Er**), Yb (**8Yb**)), (4npa = 4-nitrophenylacetate). (**1La-8Yb** = La (**1**) – Yb (**8**) in the figure).

Second weight loss in the TGA measurements

Attempts were made to fit the observed weight loss to the formation of diarylketones (Two reactions), diaryls, or an aryl ester, as these are possible products observed on decomposition of electropositive metal carboxylates [3].

Possible equations:

$$2\ RE(O_2CR)_3 \longrightarrow RE_2O(O_2CR)_4 + R_2CO + CO_2 \quad (1)$$

$$4\ RE(O_2CR)_3 \longrightarrow RE_4(O_2CR)_6(C_2O_4)_3 + 3\ R_2 \quad (2)$$

$$2\ RE(O_2CR)_3 \longrightarrow RE_2(O_2CR)_4(CO_3) + R_2CO \quad (3)$$

It was found (Table S10) that the majority of weight losses corresponded to expected values for formation of a ketone either with concomitant formation of a carbonato species or of an oxido species and CO2. Two were best fitted to diaryl formation, but it is plausible that these also give ketones by with a less clear end point.

Table S10. TGA weight loss percentages around 110 – 265 or 300 °C for compounds **1-8**.

Compound	Weight loss % from TGA	Calculated value of removing R_2CO (%) (Eq. 3)	Calculated value of removing $3R_2$ (%) (Eq. 2)	Calculated value of removing $R_2CO + CO_2$ (%) (Eq. 1)
$\{[La(4npa)_3(H_2O)_2]\cdot 2H_2O\}_n$ (**1La**)	25.0 (110 - 350 °C)	20.0	27.2	22.9
$\{[Nd(4npa)_3(H_2O)_2]\cdot 2H_2O\}_n$ (**2Nd**)	26.5 (110 - 350 °C)	19.8	27.0	22.7
$[Ce(4npa)_3(H_2O)_2]_n$ (**3Ce**)	20.4 (110 - 350 °C)	21.0	28.5	24.0
$\{[Gd_2(4npa)_6(H_2O)]\cdot 2H_2O\}_n$ (**4Gd**)	25.7 (110 - 380 °C)	20.7	28.2	23.8
$\{[Dy_2(4npa)_6(H_2O)]\cdot 2H_2O\}_n$ (**5Dy**)	24.4 (110- 380 °C)	20.6	28.0	23.6
$\{[Y_2(4npa)_6(H_2O)]\cdot 2H_2O\}_n$ (**6Y**)	27.2 (200 - 400 °C)	22.9	31.1	26.2
$\{[Er_2(4npa)_6(H_2O)]\cdot 2H_2O\}_n$ (**7Er**)	22.3 (110 - 375 °C)	20.4	27.8	23.4
$\{[Yb_2(4npa)_6(H_2O)]\cdot 2H_2O\}_n$ (**8Yb**)	27.2 (220 - 375 °C)	20.3	27.6	23.2

References

1. Sheldrick, G.M. Crystal structure refinement with SHELXL. *Acta. Crystallogr. C. Struct. Chem.* **2015**, *71*, 3–8. https://doi.org/10.1107/S2053229614024218
2. Dolomanov, O.V.; Bourhis, L.J.; Gildea, R.J.; Howard, J.A.K.; Puschmann, H. OLEX2: a complete structure solution, refinement and analysis program. *J. Appl. Crystallogr.* **2009**, *42*, 339–341. https://doi.org/10.1107/S0021889808042726
3. Deacon, G. B. Synthesis of Organometallic Compounds by Thermal Decarboxylation. *Organomet. Chem. Rev A*, **1970**, *5*, 355-372.
